# Metabolomics Approach Identifies Predictive Serum Markers for Hepatocellular Carcinogenesis Following Hepatitis C Virus Elimination

**DOI:** 10.3390/cancers18122003

**Published:** 2026-06-20

**Authors:** Takeshi Chida, Satoshi Sakai, Masahiko Ito, Kazumasa Sekihara, Kazuyoshi Ohta, Masahiro Matsushita, Gou Murohisa, Fujito Kageyama, Yuzo Sasada, Tatsuki Oyaizu, Minoru Tsugiki, Katsutoshi Tamakoshi, Tomomi Okubo, Sachiyo Yoshio, Masanori Atsukawa, Akihito Tsubota, Yasuhito Tanaka, Tatsuya Kanto, Toshiyuki Ojima, Kazuhito Kawata, Takafumi Suda, Tetsuro Suzuki

**Affiliations:** 1 Second Department of Internal Medicine, Hamamatsu University School of Medicine, 1-20-1 Handayama, Hamamatsu 431-3192, Shizuoka, Japan; 2 Department of Molecular Biology, Hamamatsu University School of Medicine, 1-20-1 Handayama, Hamamatsu 431-3192, Shizuoka, Japan; ssakai@hama-med.ac.jp; 3 Department of Microbiology and Immunology, Hamamatsu University School of Medicine, 1-20-1 Handayama, Hamamatsu 431-3192, Shizuoka, Japantesuzuki@hama-med.ac.jp (T.S.); 4 Department of Disease Model, Research Institute for Radiation Biology and Medicine, Hiroshima University, 1 Chome-3-2 Kagamiyama, Higashihiroshima 734-8553, Hiroshima, Japan; 5 Department of Gastroenterology, Shimada General Medical Center, 1200-5 Noda, Shimada 427-8501, Shizuoka, Japan; 6 Department of Hepatology, Seirei Hamamatsu General Hospital, 2-12-12 Sumiyoshi, Hamamatsu 430-8558, Shizuoka, Japan; 7 Department of Hepatology, Hamamatsu Medical Center, 328 Tomitsuka, Hamamatsu 432-8580, Shizuoka, Japan; 8 Department of Hepatology, Iwata City Hospital, 512-3 Okubo, Iwata 438-8550, Shizuoka, Japan; 9 Department of Gastroenterology, Shizuoka City Shizuoka Hospital, 10-93 Otemachi, Shizuoka 420-8630, Shizuoka, Japan; 10 Minoru Medical Clinic, Mishima 1784-1, Hamamatsu 430-0853, Shizuoka, Japan; 11 Tamakoshi Clinic, Maruzuka 262-1, Hamamatsu 435-0046, Shizuoka, Japan; 12 Division of Gastroenterology, Department of Internal Medicine, Nippon Medical School Chiba Hokusoh Hospital, 1715 Kamagari, Inzai 270-1694, Chiba, Japan; 13 The Research Center for Hepatitis and Immunology, National Center for Global Health and Medicine, 1-7-1 Kohnodai, Ichikawa 272-8516, Chiba, Japankanto.t@jihs.go.jp (T.K.); 14 Division of Gastroenterology and Hepatology, Department of Internal Medicine, Nippon Medical School, 1-1-5 Sendagi, Tokyo 113-8602, Japan; 15 Project Research Units, Research Center for Medical Science, The Jikei University School of Medicine, 3-25-8 Nishi-Shimbashi, Tokyo 105-8461, Japan; atsubo@jikei.ac.jp; 16 Department of Gastroenterology and Hepatology, Faculty of Life Sciences, Kumamoto University, 1-1-1 Honjo, Kumamoto 860-8555, Kumamoto, Japan; 17 Department of Community Health and Preventive Medicine, Hamamatsu University School of Medicine, 1-20-1 Handayama, Hamamatsu 431-3192, Shizuoka, Japan

**Keywords:** hepatocellular carcinoma (HCC), hepatitis C virus (HCV), metabolomics, sustained virological response (SVR)

## Abstract

**Background**: Abnormalities in energy and amino acid metabolism are potentially involved in hepatocellular carcinoma (HCC) development. This study aimed to identify serum metabolites predictive of HCC following sustained virological response (SVR) with hepatitis C virus (HCV) treatment. **Methods**: Comparative metabolomics was conducted using time-course serum samples from patients who failed interferon-based therapy but subsequently achieved SVR with direct-acting antivirals (DAAs), minimizing inter-individual variability. Predictive biomarkers for post-SVR HCC were extracted from the results and validated by comparing 29 patients who developed post-SVR HCC with 58 age-matched patients who remained HCC-free during follow-up. **Results**: Metabolite concentrations changed more markedly after treatment in SVR cases than in non-SVR cases. Significant changes in methionine (Met), methionine sulfoxide (MetO), and ornithine (Orn) levels before and after treatment (Pre- and Post-Tx) were found only in the non-HCC group. Regression and survival analyses identified high levels of Pre- and Post-Tx Orn, Pre-Tx Met, and Post-Tx MetO as predictors of post-SVR HCC and enabled risk stratification. The integration of these metabolites with the fibrosis-4 (FIB-4) index and alpha-fetoprotein (AFP) facilitated risk stratification and discriminated between high- and low-risk patients. The Pre-Tx FIB-4/Met model and the Post-Tx AFP/MetO/Orn model identified low- and high-risk groups with 3-year HCC incidence rates of 6.4% and 81.8%, respectively. **Conclusions**: Serum Met, MetO, and Orn were identified as candidate biomarkers associated with post-SVR HCC development, which remains a concern in the fight against hepatitis C. Combining these metabolites with established clinical markers may improve post-SVR HCC risk stratification.

## Simple Summary

Hepatocellular carcinoma (HCC) remains a clinically important complication in a subset of patients even after sustained virological response (SVR) is achieved for hepatitis C virus infection, thus necessitating more accurate methods for identifying those at continued risk. This study was designed to determine whether serum metabolites measured before and after SVR could predict post-SVR HCC development. Methionine, methionine sulfoxide, and ornithine were identified as metabolites associated with subsequent HCC risk, and their combination with established clinical markers improved discrimination between low- and high-risk patients. These findings suggest the potential utility of metabolite-based risk stratification for post-SVR HCC and may contribute to more individualized surveillance strategies following SVR.

## 1. Introduction

Hepatitis C virus (HCV) infection is a well-established cause of hepatocellular carcinoma (HCC), which is the most serious consequence of chronic HCV infection [1,2]. Even after patients with persistent HCV infection achieve sustained virological response (SVR) with chemotherapy, the risk of developing HCC is not completely excluded. The incidence of HCC tends to be particularly high in hepatitis C patients with persistent adverse conditions such as metabolic disorders and progressive fibrosis. Despite the substantial progress made in viral clearance through the development of direct-acting antivirals (DAAs) over the past few decades—achieving SVR rates over 95% across all HCV genotypes—the risk of developing HCC following SVR remains between 0.9 and 2.96 per 100 patients each year, leaving a significant number of patients still at risk for HCC [3,4].

Although the importance of regular screening for the prevention of HCC in patients who have achieved SVR is widely recognized, standard guidelines for post-SVR HCC surveillance have not yet been established worldwide. To establish appropriate surveillance programs for the early detection of HCC, it is also important to avoid excessive testing of patients at low HCC risk while considering healthcare economics and patient burden. Therefore, rather than managing all patients uniformly, it is crucial to stratify the risk of developing HCC through accurate assessment of SVR patients based on scientific evidence and to establish screening protocols for each risk level [5]. The modality and frequency of HCC screening should be customized according to an individual’s risk profile, taking into account cost-effectiveness [2,6].

Several studies have explored factors that may be useful in predicting HCC development after achieving SVR. Liver function and fibrosis markers have been most intensively studied [7,8], and although a high fibrosis-4 (FIB-4) index can be a predictor of HCC, it is not always clear whether the risk of HCC decreases as the FIB-4 index decreases due to a significant improvement in aspartate aminotransferase (AST) and alanine aminotransferase (ALT) levels associated with the resolution of inflammation after SVR [9]. Alpha-fetoprotein (AFP) is most commonly used as a marker for HCC in Japan [4,10], but it is not included as a biomarker for the diagnosis or prognosis of HCC in European or American guidelines. Noninvasive assessments of liver stiffness have been developed, such as shear wave elastography and magnetic resonance elastography [11,12], but the accuracy of prediction based on stiffness alone is limited because HCC can occur in patients without increased liver stiffness. Other factors such as abnormal glucose and lipid metabolisms and excessive alcohol intake are also involved in the development of HCC. Since the mechanisms underlying the development of HCC after SVR are multifactorial, it is generally accepted that accurate prediction of HCC after SVR requires the establishment of a reliable, scientifically based prediction model consisting of a combination of multiple biomarkers [13]. This underscores the requirement to identify biomarkers applicable to diverse patient backgrounds, ideally those that provide additional predictive value beyond fibrosis markers.

Persistent HCV infection is known to cause abnormalities in various metabolic processes, such as lipid, sugar, and iron. These metabolic abnormalities have been suggested to induce a stress response in the host liver, thus leading to progressive fibrosis, angiogenesis, and cell proliferation and ultimately contributing to carcinogenesis [14]. Although, in many cases, metabolic abnormalities resulting from HCV infection are expected to improve to some extent after SVR, irreversible metabolic disturbances may persist with the progression of liver fibrosis and (epi-)genetic changes that occurred during chronic infection [15]. Given that persistent metabolic abnormalities in the liver may be associated with the development of HCC, it is plausible that certain metabolites that are not properly regulated after SVR may be biomarkers that predict liver carcinogenesis. Metabolomics enables a comprehensive analysis of metabolites in biological fluids and has been used in the diagnosis and prognosis of several cancers, including HCC [16,17]. Although previous studies have identified distinctive metabolic profiles in HCC patients [18,19], the metabolic alterations associated with post-SVR HCC development remain incompletely understood. The aim of this study is to identify biomarkers from serum metabolites that may be beneficial for stratifying the risk of developing HCC after SVR in HCV-infected patients.

## 2. Materials and Methods

### 2.1. Study Design, Population, and Sample Collection

Supplementary Table S1 summarizes the characteristics of the enrolled patients. For Experiments (Exp) 1 and 2, a total of 4 and 11 patients with chronic HCV infection who were treated with an interferon-based regimen were recruited from Hamamatsu University Hospital, respectively. Serum samples were collected under fasting conditions at the time points indicated in Supplementary Tables S2 and S3. For Exp 3 and 4, a total of 17 non-SVR patients and 29 patients who developed HCC after SVR with interferon-free (IFN-free) DAA regimens were recruited from 13 affiliated institutions. These analyses were designed as internal matched-comparison studies using patients derived from the same source population. For these analyses, patients in the SVR group and those in the non-HCC group were selected as controls and matched 2:1 by age to the non-SVR and HCC groups, respectively; the corresponding standardized mean differences for age were 0.033 and 0.062. Serum samples were collected before treatment initiation (Pre-Tx) and 24 weeks after treatment completion (Post-Tx) and immediately stored at −80 °C. The study protocols conformed to the ethical guidelines of the Declaration of Helsinki. All participating patients provided written informed consent.

### 2.2. Patient Follow-Up and Exclusion Criteria

SVR was defined as the absence of detectable HCV RNA at 24 weeks after treatment completion. All patients were followed up at intervals of 1–6 months for biochemical and virological values. Imaging examinations (ultrasonography [US], contrast-enhanced computed tomography [CT], or contrast-enhanced magnetic resonance imaging [MRI]) were performed at least once every 6 months. A diagnosis of HCC development was based on findings of typical vascular patterns on contrast-enhanced CT or MRI.

The exclusion criteria were as follows: (1) the absence of sufficiently stored serum samples; (2) coinfection with either hepatitis B virus (HBV) or human immunodeficiency virus; (3) a history of other chronic liver diseases (autoimmune hepatitis, primary biliary cholangitis, hemochromatosis, or Wilson’s disease); (4) the presence of viable HCC from enrollment up to 24 weeks after treatment completion; and (5) viral relapse (except for the non-SVR group).

### 2.3. Metabolome Analyses

Fifty microliters of the serum was added to 450 µL of methanol containing an internal standard (IS) solution (Human Metabolome Technologies (HMT), Tsuruoka, Japan) at 0 °C. The solution was thoroughly mixed and centrifuged at 2300× *g* for 5 min at 4 °C. The aqueous phase was filtered through a 5 kDa cutoff filter by centrifugation at 9100× *g* for 6 h to remove macromolecules. The filtrate was desiccated and dissolved in 25 µL of Milli-Q water for the subsequent analyses. Metabolome analyses were performed according to HMT’s Basic Scan package, using capillary electrophoresis time-of-flight mass spectrometry (CE-TOFMS) with an Agilent CE capillary electrophoresis system (Agilent Technologies, Santa Clara, CA, USA) as previously described [20]. The system was connected via a fused silica capillary (50 μm i.d. × 80 cm total length) with electrophoresis buffers (H3301-1001 for cation analysis and I3302-1023 for anion analysis; HMT) as the electrolyte.

### 2.4. Liquid Chromatography Tandem Mass Spectrometry (LC-MS/MS)

The details of the sample preparation method are provided in the Supplementary Materials. The LC system included Acquity UPLC (Waters, Milford, MA, USA) and a Discovery HS F5 HPLC column (150 mm length × 2.1 mm i.d., particle size 3 μm; Sigma-Aldrich, St. Louis, MO, USA). MS/MS analysis was conducted in positive ESI mode using a 4000Q-TRAP mass spectrometer (AB SCIEX, Framingham, MA, USA). Measurement conditions and representative peak profiles are detailed in Supplementary Table S4 and Figure S1.

### 2.5. Data Analyses

Multivariate pattern recognition analyses were carried out using MetaboAnalyst 6.0 software (http://www.metaboanalyst.ca/, last accessed on 13 September 2024) [21]. Statistical analyses were performed using SPSS ver. 25.0 (SPSS, Chicago, IL, USA), GraphPad Prism (ver. 9.0; GraphPad Software, San Diego, CA, USA), EZR (ver 1.60; Saitama Medical Center, Jichi Medical University, Saitama, Japan), and R software (version 4.6.0; R Foundation for Statistical Computing, Vienna, Austria). The Mann–Whitney U and Wilcoxon signed-rank tests were used to evaluate continuous variables and paired data, respectively. The Cox proportional hazards model was used for uni- and multivariate analyses. All variables were entered into the Cox proportional hazards models as continuous variables. No log-transformation or standardization was applied before Cox regression analyses. Cumulative incidence was evaluated using the Kaplan–Meier method. The receiver operating characteristic (ROC) curves were used to determine the optimal cutoff values, which were derived and evaluated within the same cohort. A competing risk analysis was not performed in the present study. PLS-DA models were evaluated using cross-validation and permutation testing. Model performance and validity were assessed using R2Y(cum), Q2(cum), and permutation test *p*-values (pR2Y and pQ2). Multiple-testing correction for metabolite comparisons was performed using the Benjamini–Hochberg false discovery rate (FDR). Bootstrap resampling analyses were performed to assess the selection stability of candidate metabolites. The Cox prediction models were internally validated using bootstrap optimism correction with 1000 bootstrap resamples. Model discrimination was evaluated using the concordance index (C-index). Calibration plots comparing predicted and observed 3-year HCC risks were generated using bootstrap resampling.

## 3. Results

### 3.1. Dynamic Changes in Serum Metabolic Profiles with HCV Clearance

**Figure 1:**
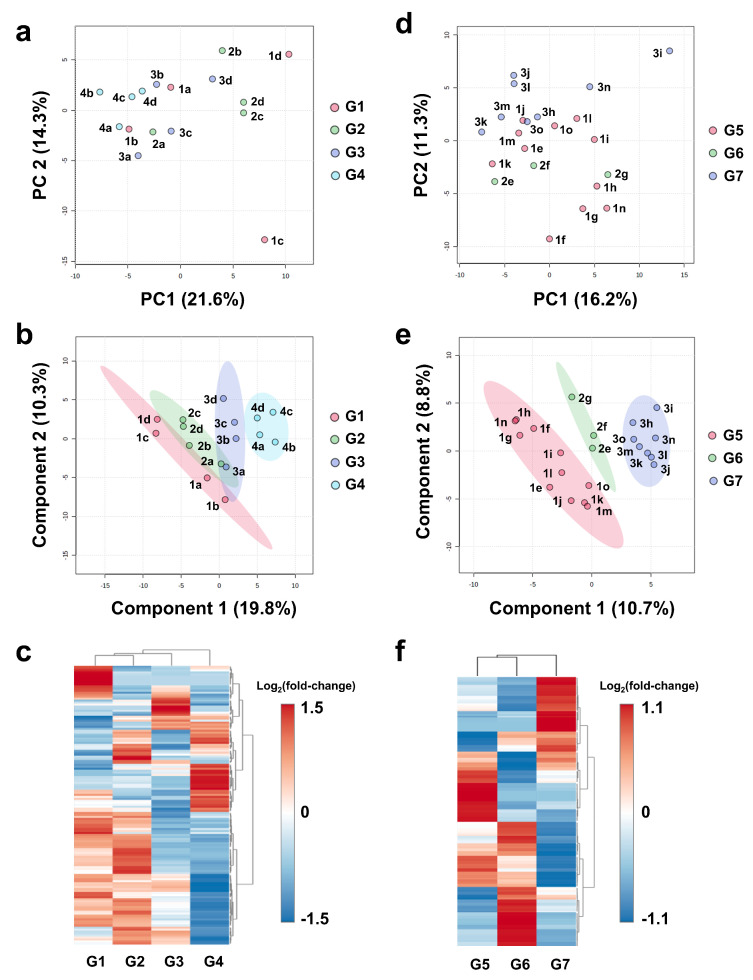
Dynamics in serum metabolic profiles with or without HCV clearance. Metabolome analysis results for Exp 1 (panels (**a**–**c**)) and Exp 2 (panels (**d**–**f**)) are presented. Each sample was identified by labels as 1a, 2a, etc. (**a**–**c**) G1: pre-1st treatment, G2: post-1st treatment without viral clearance, G3: pre-2nd treatment, and G4: post-2nd treatment with viral clearance. (**d**–**f**) G5: pre-treatment, G6: post-treatment without viral clearance, and G7: post-treatment with viral clearance. (**a**,**d**) Score plots of principal component analysis. (**b**,**e**) Score plots of partial least squares discriminant analysis. The 95% confidence regions are displayed using colored ellipses. (**c**,**f**) Heat maps created from hierarchical clustering. Each column represents a group and each row represents a metabolite for which significant difference was observed. The color intensity of each metabolite was scaled according to log_2_(fold-change) values. Abbreviations: Exp, experiment; PC, principal component.

In Experiment 1, four cases (patients a–d) were selected in which the same patient received two rounds of antiviral therapy, the first treatment (pegylated interferon-α plus ribavirin; PR) was ineffective, and SVR was achieved with the second (Telaprevir plus PR; TPR) (Supplementary Table S1—Exp 1 and Supplementary Table S2). All serum samples were grouped according to the time point at which they were collected: before the first treatment (G1), without viral disappearance after the first treatment (G2), before the second treatment (G3), and with viral clearance after the second treatment (G4). Semi-quantitative, non-targeted metabolomics using CE-TOFMS identified 148 metabolites (water-soluble and ionizable metabolites including sugars, amino acids, nucleic acids, organic acids, and short- and medium-chain fatty acids) from each serum. Principal component analysis (PCA) performed on the obtained measurements and patient information visualized the differences in metabolic profiles among all specimens: the G4 samples tended to show negative scores along PC1, while the G1–G3 samples showed no distinctive features that distinguished them from the others (Figure 1a). Euclidean distances from G1 were calculated for the other groups based on PCA, indicating that G1–G4 showed significantly greater distances than G1–G2 and a longer distance than G1–G3 (Supplementary Figure S2a). Further characterization of the differences between groups using partial least squares discriminant analysis (PLS-DA) showed that the 95% confidence ellipses for G2 and G3 overlapped with those for G1 but not for G4. The G4 samples were shown to be the farthest from the G1 samples (Figure 1b). The PLS-DA model showed R2Y(cum) = 0.902 and Q2(cum) = 0.385. Permutation testing demonstrated borderline significance (pR2Y = 0.246, pQ2 = 0.057). Hierarchical cluster analysis (HCA), displayed as a dendrogram with heat map based on Euclidean distance and Ward’s method, showed that G1 and G2 were closely clustered, with G4 having the most different metabolic profile from the other three groups (Figure 1c). Samples G1, G2, and G3 were collected during the HCV-positive period, whereas G4 was collected after viral clearance. Notably, metabolomic profiles remained relatively similar across the three HCV-positive time points despite different treatment phases, whereas a marked shift was observed only after SVR. Because these comparisons were performed serially within the same individuals, the observed metabolic alterations are unlikely to be attributable to inter-individual variability and instead support an association with SVR. The relatively similar profiles of G1 and G2 further suggest that the effect of drug administration itself on metabolite concentrations was limited.

Further metabolomic analysis (Experiment 2) was performed using sera collected before and after treatment for patients who achieved or failed to achieve SVR in the first-time treatment with TPR, and 185 metabolites were identified from each serum sample (patient characteristics are summarized in Supplementary Table S1—Exp 2 and Supplementary Table S3). The pre-treatment samples of all patients, the post-treatment samples of the three patients who failed to achieve SVR (patients e–g), and the samples of the eight patients who achieved SVR (patients h–o) were classified as G5, G6, and G7, respectively. PCA visualized inter-sample differences in metabolite profiles, with G7 samples having positive PC2 scores, while G6 samples had negative scores (Figure 1d). In terms of Euclidean distance, G5–G7 was more distant than G5–G6 (Supplementary Figure S2b). PLS-DA showed that 95% confidence ellipses for all groups did not overlap, and G7 was more distant from G5 than G6 (Figure 1e). The PLS-DA model showed R2Y(cum) = 0.945 and Q2(cum) = 0.353. Permutation testing demonstrated significant model validity (pR2Y = 0.015, pQ2 = 0.023). HCA visually showed a gap between G7 and the other two groups (Figure 1f). These results emphasize that the changes in metabolite concentrations after achieving SVR are more pronounced than in non-SVR cases, suggesting that HCV clearance has a significant impact on the metabolic profile.

### 3.2. Selection of Candidate Metabolites Whose Serum Levels Change Significantly After HCV Clearance and May Be Involved in Liver Pathophysiology

Loading scatter plots were created from PCA data from Exp 1 and 2 to find the serum metabolites that have a pronounced impact on the characterization of the metabolic profile of each sample group (Supplementary Figure S3a,b). In these plots, metabolites located away from the zero origin of the PC1 and PC2 axes, i.e., at the periphery of the plots, show their contribution to data separation and specificity in profiling. Considering the plot patterns obtained and the involvement of metabolites in liver pathophysiology, methionine (Met), methionine sulfoxide (MetO), hydroxyproline (Hyp), carnitine (Car), ornithine (Orn), asymmetric dimethylarginine (ADMA), arginine (Arg), and acetyl-carnitine (ACar) were selected as candidate metabolites showing marked changes in serum concentrations after HCV clearance. Given that only a limited number of metabolites remained statistically significant after FDR correction, candidate metabolites were selected based on a combination of loading plot characteristics and biological relevance rather than statistical significance alone. Bootstrap resampling analyses further demonstrated higher selection stability for methionine sulfoxide than the other candidate metabolites (Supplementary Table S5).

### 3.3. Identification of Metabolites Whose Serum Concentrations Changed Significantly with Interferon-Free DAA Treatment in SVR Group but Not in the Non-SVR Group

Experiment 3 (Supplementary Table S1—Exp 3) was designed as an internal matched-comparison study to evaluate metabolite changes before and after DAA therapy in patients who achieved SVR and those who did not. The serum levels of these metabolites were quantitatively measured before and after treatment with an interferon-free DAA regimen (Tx) and between SVR and non-SVR using the LC-MS/MS multiple reaction monitoring system (Supplementary Figure S1, Supplementary Table S4). In the SVR group, Post-Tx values of AST, ALT, AFP, ALBI score, and FIB-4 index were significantly lower than Pre-Tx values, while albumin levels were significantly higher. In contrast, in the non-SVR group, only AST, AFP, and FIB-4 index decreased significantly with treatment (Supplementary Tables S6 and S7).

**Figure 2:**
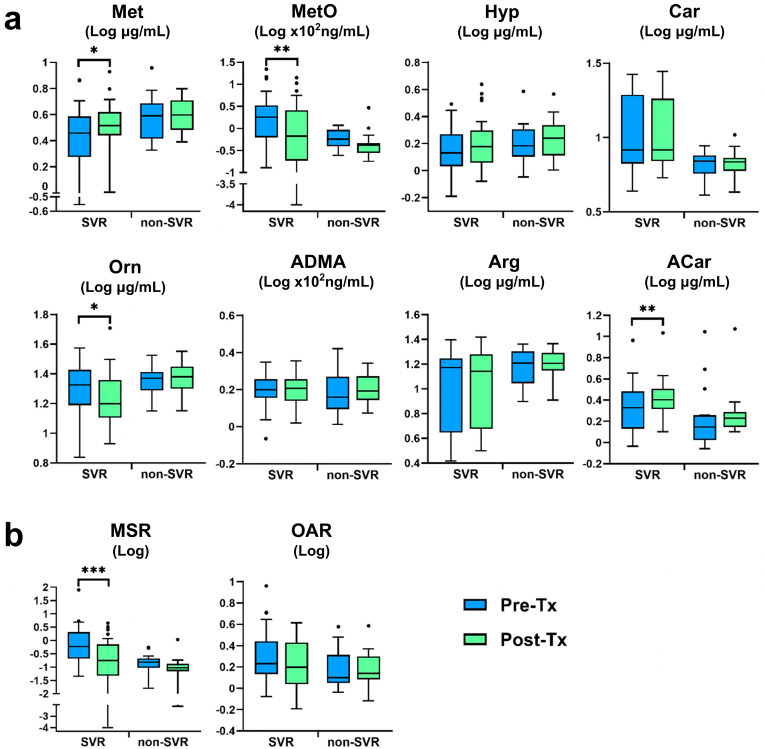
Serum metabolites in the group of patients achieving SVR and that of non-SVR patients following DAA treatment. Comparison of (**a**) serum metabolites and (**b**) specific metabolite ratios at Pre- and Post-Tx in each group. Boxplots represent the interquartile range (IQR); median is indicated by the horizontal line inside the box. Whiskers extend from the lower and upper quartiles to the minimum and maximum values within 1.5 times the IQR, respectively. Outliers beyond the whiskers are plotted individually as dots. The numbers of analyzed patients were non-SVR, *n* = 17, and SVR, *n* = 34. Wilcoxon signed-rank test; * *p* < 0.05, ** *p* < 0.01, and *** *p* < 0.001. Abbreviations: ACar, acetyl-carnitine; ADMA, asymmetric dimethylarginine; Arg, arginine; Car, carnitine; Hyp, hydroxyproline; IQR, interquartile range; Met, methionine; MetO, methionine sulfoxide; MSR, methionine–methionine sulfoxide ratio; OAR, ornithine–arginine ratio; Orn, ornithine; SVR, sustained virological response.

Serum metabolite concentrations at Pre- and Post-Tx are shown in Figure 2a and Supplementary Table S7. Although DAAs, especially NS3 protease inhibitors, are known to have the potential to affect lipid and uric acid metabolism, the effects of DAAs on serum metabolites were considered negligible at the time of blood collection (24 weeks after treatment ended) since the blood half-life of the NS3 inhibitors used here is approximately 8 to 20 h [22]. In the SVR group, Post-Tx values for Met and ACar were significantly higher than Pre-Tx values, 1.19-fold for Met and 1.19-fold for ACar, respectively; MetO and Orn levels decreased significantly after treatment, 0.61- and 0.88-fold for MetO and Orn, respectively. In contrast, in the non-SVR group, there were no significant differences in the levels of these metabolites before and after treatment. Since the levels of MetO and Orn depend on the levels of their substrates (Met and Arg) and their relative ratios to the substrates are important for pathophysiological assessment, methionine–methionine sulfoxide ratio (MSR) and ornithine–arginine ratio (OAR) were also calculated. In the SVR group, while OAR showed no significant difference between Pre- and Post-Tx, MSR at Post-Tx was significantly reduced by 0.19-fold compared to that at Pre-Tx (Figure 2b).

Thus, Post-Tx levels of Met, MetO, Orn, and ACar were significantly altered in the SVR group compared to their Pre-Tx levels but not in the non-SVR group, suggesting that these metabolites may reflect hepatic pathology and/or metabolic changes associated with HCV clearance.

### 3.4. Dynamics of Serum Metabolites Associated with Development of HCC After Achieving SVR

**Figure 3:**
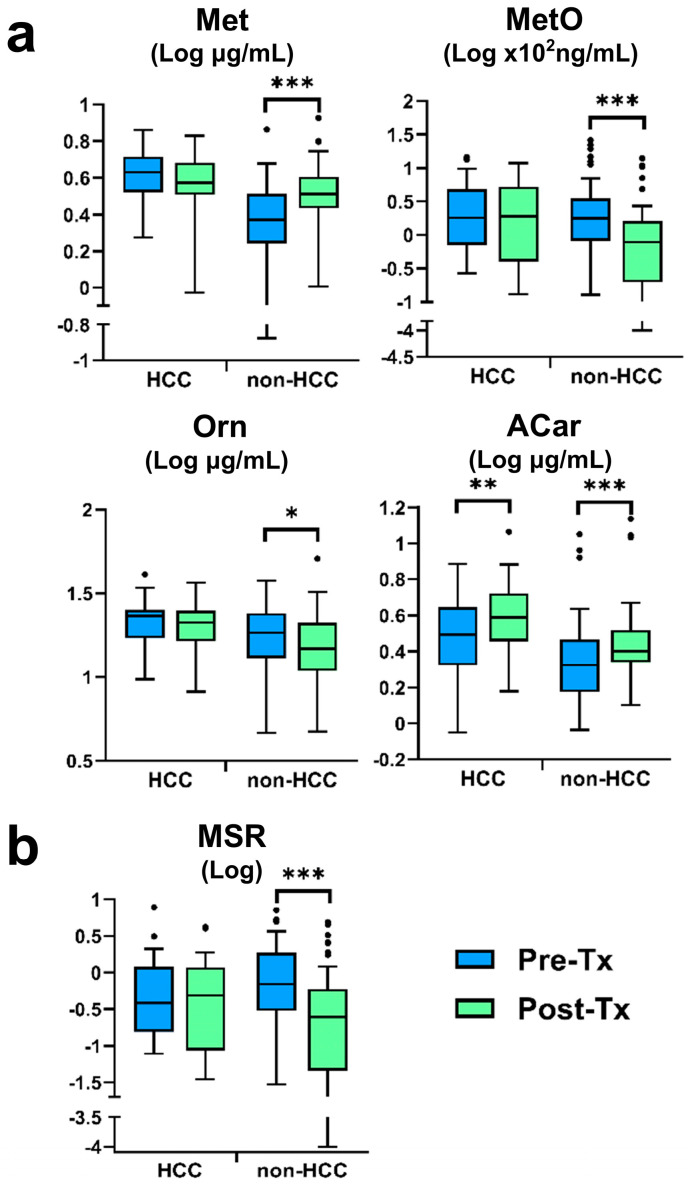
Serum metabolites in the groups of patients with or without HCC development after achieving SVR. Comparison of (**a**) serum metabolites and (**b**) methionine–methionine sulfoxide ratio at Pre- and Post-Tx in each group. The boxplots and whiskers are shown as described in the legend to Figure 2. The numbers of analyzed patients were HCC, *n* = 29, and non-HCC, *n* = 58. Wilcoxon signed-rank test; * *p* < 0.05, ** *p* < 0.01, and *** *p* < 0.001. Abbreviations: ACar, acetyl-carnitine; HCC, hepatocellular carcinoma; Met, methionine; MetO, methionine sulfoxide; MSR, methionine–methionine sulfoxide ratio; Orn, ornithine; SVR, sustained virological response.

Although the SVR group showed overall changes in metabolite levels between Pre- and Post-Tx, these changes were not uniformly observed across individual patients (Figure 2). To further investigate whether post-treatment metabolic profiles were associated with subsequent HCC development after SVR, Experiment 4 (Supplementary Table S1—Exp 4) was designed as an internal matched-comparison study. Patients who developed HCC within 5 years after achieving SVR (HCC group; *n* = 29) were compared with patients who achieved SVR but did not develop HCC (non-HCC group; *n* = 58). No significant differences were observed in body mass index, alcohol intake, IFN treatment history, HCV serogroup, treatment regimen, or Pre-Tx HCV viral load between the two groups (Supplementary Table S8). The rates of pre-existing diabetes and HCC were higher in the HCC group. Compared to Pre-Tx, Post-Tx AST, ALT, and AFP levels, as well as ALBI score and FIB-4 index, were significantly lower, while albumin level and platelet counts were significantly higher in both groups (Supplementary Table S9). Regarding serum levels of Met, MetO, Orn, and ACar, Post-Tx levels of Met and ACar were significantly higher than Pre-Tx levels in the non-HCC group, with Met increasing 1.36-fold and ACar 1.23-fold. In contrast, Post-Tx levels of MetO and Orn were significantly lower, decreasing 0.45-fold for MetO and 0.87-fold for Orn (Figure 3a and Supplementary Table S9). In the HCC group, the only metabolite change was a 1.24-fold increase in ACar. With respect to metabolite ratios, the Post-Tx level of MSR in the non-HCC group was markedly reduced by 0.09-fold compared to Pre-Tx levels (Figure 3b).

It is thus interesting to note that Pre- and Post-Tx changes in Met, MetO, Orn, and ACar concentrations in the non-HCC group were consistent with the characteristics seen in the SVR group, whereas Met, MetO, and Orn concentrations in the HCC group did not show such a significant change. MSR also showed significant changes in the non-HCC group, whereas the differences between Pre- and Post-Tx were only limited in the HCC group.

### 3.5. Serum Metabolites as Predictors of HCC Development After Achieving SVR

Univariate analysis was performed to evaluate whether the selected metabolites and MSR could serve as candidate biomarkers associated with subsequent HCC development after achieving SVR. The significance of Met, MetO, Orn, ACar, and MSR was examined as independent variables, as well as AFP and FIB-4 index, which are known to be associated with post-SVR HCC development [10,23,24]. Both Pre- and Post-Tx measurements of Orn and ACar, Pre-Tx measurements of Met, and Post-Tx measurements of MetO were identified as significant risk factors for the development of HCC after achieving SVR, and Post-Tx measurements of AFP and Pre- and Post-Tx measurements of FIB-4 index were also associated with the development of HCC after achieving SVR (Table 1).

Based on the findings obtained, Met and Orn values at Pre-Tx and MetO and Orn values at Post-Tx were selected as candidate biomarkers based on the following conditions: metabolites that (1) showed significant quantitative changes at Post-Tx compared to Pre-Tx in the SVR and non-HCC groups but not in the non-SVR and HCC groups (Figure 2 and Figure 3) and (2) were identified by univariate analysis as significant risk factors for the development of HCC after SVR (Table 1).

Multivariate analysis was performed to identify independent predictors among the candidate metabolites and the established clinical markers, AFP and FIB-4 index. For Pre-Tx factors, we examined two combinations: FIB-4 index with Met and FIB-4 index with Orn. In both models, both factors remained independently associated with HCC development after achieving SVR (Table 2, upper panel, Models 1 and 2). Confounding with Orn was observed in the analysis combining all three factors (Model 3). In contrast, when Post-Tx levels of MetO or Orn were combined with either FIB-4 index or AFP levels, both factors were identified as independent risks in all combinations (Table 2, bottom row, Models 1–4). Confounding between FIB-4 index and AFP was observed in Model 5; when the three factor combinations of Met and Orn plus either FIB-4 index or AFP were analyzed, each factor was identified as an independent risk in all combinations (Models 6 and 7).

To further evaluate model performance, internal validation analyses were performed for the principal prediction models. Bootstrap optimism correction demonstrated acceptable discrimination performance across the evaluated Cox models. The optimism-corrected C-index was 0.702 for Pre-Tx FIB-4 index alone and 0.775 for Pre-Tx FIB-4 index and Met; 0.646 for Post-Tx FIB-4 index alone and 0.725 for Post-Tx FIB-4 index, MetO, and Orn; and 0.758 for Post-Tx AFP alone and 0.794 for Post-Tx AFP, MetO, and Orn. These findings suggest that the addition of metabolite markers improved model discrimination compared with established clinical markers alone (Supplementary Table S10). Calibration plots showed generally acceptable agreement between predicted and observed 3-year HCC risks, although the estimates should be interpreted cautiously because of the limited number of events (Supplementary Figure S4).

### 3.6. Stratification of Risk of Developing HCC After Achieving SVR by Serum Metabolites and Liver Function Tests

**Figure 4:**
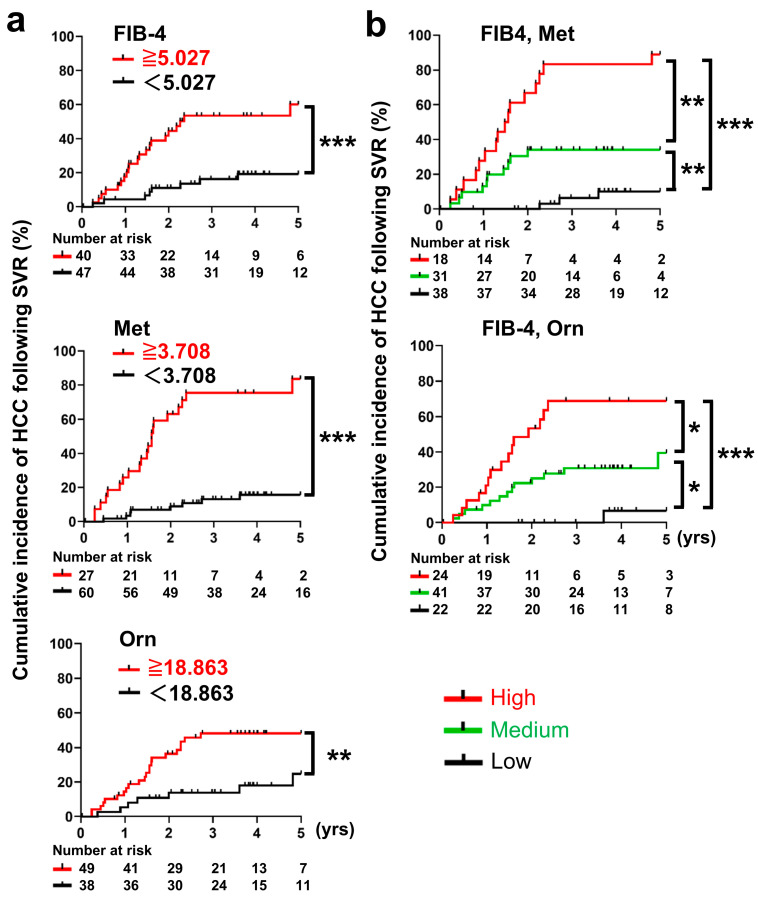
Cumulative incidences of HCC in patients achieving SVR stratified by independent risk factors: Pre-Tx serum metabolite levels and/or liver function test results. The optimal cutoff point for each factor in predicting HCC was defined as the point at which the mean of sensitivity and specificity were maximized. The time (years) was measured from the point at which SVR was confirmed, namely, 24 weeks after the end of treatment. (**a**) Stratification was performed by single factors: FIB-4 index ≥ 5.027, serum Met levels ≥ 3.708 µg/mL, and serum Orn levels ≥ 18.863 µg/mL. The red and black lines represent the positive or negative groups for the risk factor, respectively. (**b**) Combined factors: FIB-4 index ≥ 5.027 and serum Met levels ≥ 3.708 µg/mL, and FIB-4 index ≥ 5.027 and serum Orn levels ≥ 18.863 µg/mL. The red, green and black lines represent the high-risk group (positive for two factors), medium-risk group (positive for one factor), and low-risk group (neither), respectively. Cumulative incidences were assessed using the Kaplan–Meier method and log-rank test. The ‘Number at risk’ indicates the number of subjects remaining at each time point who are still at risk of developing HCC. Log-rank test; * *p* < 0.05, ** *p* < 0.01, and *** *p* < 0.001. Abbreviations: FIB-4, fibrosis 4; HCC, hepatocellular carcinoma; Met, methionine; Orn, ornithine; Pre-Tx, pre-treatment; SVR, sustained virological response.

Based on the identified independent risk factors (Table 2), the cumulative incidence of HCC after achieving SVR was analyzed using the Kaplan–Meier method. Patients were divided into two groups according to their respective cutoff values determined based on the ROC curve (Supplementary Figure S5). Cumulative HCC incidence was plotted for the groups above and below the cutoff values for Met, Orn, and FIB-4 index, risk factors for Pre-Tx (Figure 4a): Met (<3.708 vs. ≥3.708 ug/mL, *p* < 0.001), Orn (<18.863 vs. ug/mL, *p* = 0.008), and FIB-4 (<5.027 vs. ≥5.027, *p* < 0.001) all showed significant differences in HCC incidence after achieving SVR.

To further evaluate risk stratification based on combinations of multiple Pre-Tx factors, patients were categorized as high- (positive for both factors), intermediate- (positive for one factor), or low-risk (negative for both factors). Of note, when stratified by only one of the Pre-Tx factors, FIB-4 index ≥ 5.027, Met ≥ 3.708 μg/mL, or Orn ≥ 18.863 μg/mL, the cumulative incidence of HCC at 3 years after achieving SVR was 53.4%, 75.3%, and 48.2% in the high-risk group, respectively. The cumulative incidence of HCC in the low-risk group was 16.2%, 13.1%, and 13.8%, respectively. In contrast, when patients were stratified using the combination of FIB-4 index and Met or Orn, incidence increased to 83.3% and 68.8% in the high-risk group and decreased to 6.4% and 0% in the low-risk group (Figure 4b).

**Figure 5:**
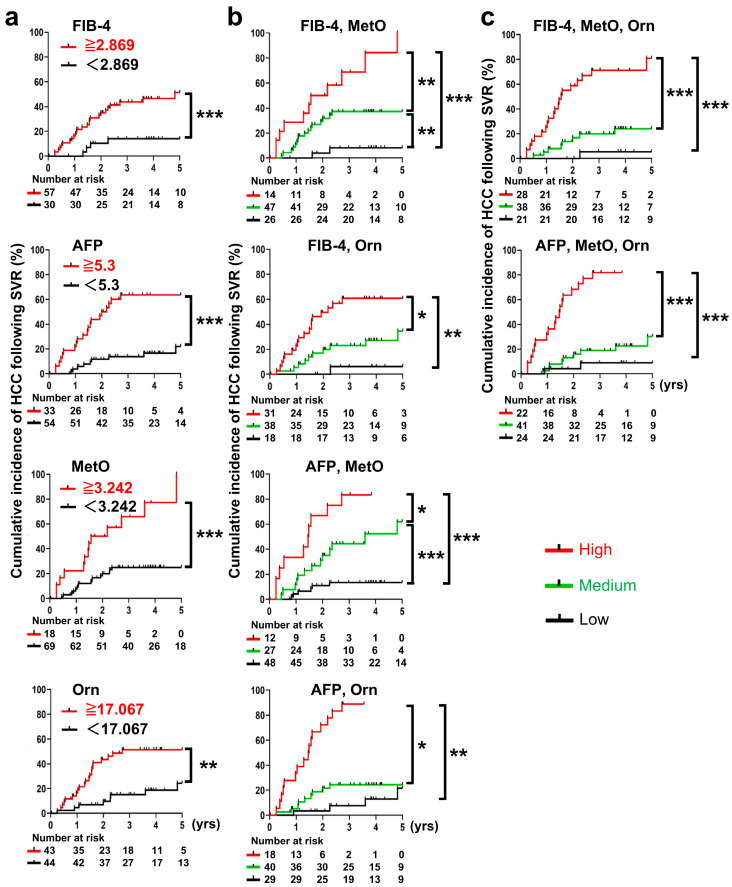
Cumulative incidences of HCC in patients achieving SVR stratified by independent risk factors: Post-Tx serum metabolite levels and/or liver function test results. The optimal cutoff point and the time point (yrs) are as described in the legend to Figure 4. (**a**) Stratification was performed by single factors: FIB-4 index ≥ 2.869, serum AFP levels ≥ 5.3 ng/mL, serum MetO levels ≥ 3.242 × 10^2^ ng/mL, and serum Orn levels ≥ 17.067 µg/mL. The red and black lines represent the positive or negative groups for the risk factor, respectively. (**b**) Combined two independent factors: FIB-4 index ≥ 2.869 and serum MetO levels ≥ 3.242 × 10^2^ ng/mL, FIB-4 index ≥ 2.869 and serum Orn levels ≥ 17.067 µg/mL, AFP levels ≥ 5.3 ng/mL and serum MetO levels ≥ 3.242 × 10^2^ ng/mL, and AFP levels ≥ 5.3 ng/mL and serum Orn levels ≥ 17.067 µg/mL. The red, green and black lines represent the high-risk group (positive for two factors), medium-risk group (positive for one factor), and low-risk group (neither), respectively. (**c**) Combined three independent factors: the combination of FIB-4 index ≥ 2.869, serum MetO levels ≥ 3.242 × 10^2^ ng/mL, and Orn levels ≥ 17.067 µg/mL, and the combination of AFP levels ≥ 5.3 ng/mL, serum MetO levels ≥ 3.242 × 10^2^ ng/mL, and Orn levels ≥ 17.067 µg/mL. The red, green and black lines represent the high-risk group (positive for two or three factors), medium-risk group (positive for one factor), and low-risk group (neither), respectively. Cumulative incidences were assessed using the Kaplan–Meier method and log-rank test. The ‘Number at risk’ indicates the number of subjects remaining at each time point who are still at risk of developing HCC. Log-rank test; * *p* < 0.05, ** *p* < 0.01, and *** *p* < 0.001. Abbreviations: AFP, alpha-fetoprotein; FIB-4, fibrosis 4; HCC, hepatocellular carcinoma; MetO, methionine sulfoxide; Orn, ornithine; Post-Tx, post-treatment; SVR, sustained virological response.

Post-Tx risk factors MetO, Orn, FIB-4 index, and AFP were also plotted as cumulative HCC incidence in the groups above and below the cutoff: MetO (<3.242 vs. ≥3.242 × 10^2^ ng/mL, *p* < 0.001), Orn (<17.067 vs. ≥17.067ug /mL, *p* = 0.001), FIB-4 (<2.869 vs. ≥2.869, *p* = 0.003), and AFP (<5.3 vs. ≥5.3 ng/mL, *p* < 0.001) all showed significant differences in HCC incidence (Figure 5a). As with the Pre-Tx factor, the two factors were stratified into three groups (high risk, intermediate risk, and low risk), and cumulative incidence was determined using the combination of the two factors. The results showed that the combination of MetO with FIB-4 or AFP and the combination of Orn with FIB-4 or AFP increased the incidence of HCC in the high-risk group and decreased the incidence in the low-risk group compared to the single-factor analysis (Figure 5b). Furthermore, when the three-factor combination of MetO and Orn plus FIB-4 or AFP was analyzed, the difference in post-SVR HCC incidence between the high- and low-risk groups was even larger than in the two-factor analysis, with the high-risk group having a higher incidence of HCC of 71.2% and 81.8%, respectively, while the low-risk group had a lower one of 5.6% and 9.0%, respectively (Figure 5c). Stratification based on the combination of FIB-4 and AFP and MetO or Orn after Tx was also performed, but these models showed relatively lower discrimination performance compared with the model shown in Figure 5c (Supplementary Figure S6).

Overall, combining the identified metabolites with FIB-4 and AFP appeared to improve risk stratification compared with the use of individual factors alone. These findings suggest that metabolite-based models may help identify patient subgroups with different post-SVR HCC risks; however, further validation is required before clinical implementation.

## 4. Discussion

Since the risk of hepatocellular carcinoma following sustained virological response is heterogeneous and influenced by comorbidities and virus-induced pathophysiological changes, post-DAA surveillance may benefit from incorporating functional biomarkers related to hepatocarcinogenesis in addition to fibrosis staging. Metabolic abnormalities involving glycolysis, the tricarboxylic acid cycle, and amino acid metabolism accompany HCC development, and HCV clearance would be expected to alleviate these disturbances [25,26]. Consistent with this concept, metabolomic profiles differed markedly before and after treatment in SVR cases, whereas such changes were not evident in non-SVR cases, suggesting partial normalization of hepatic metabolic stress following viral eradication (Figure 1 and Figure 2). Because serum metabolites were measured before treatment and 24 weeks after treatment completion but not at the time of HCC diagnosis, the identified metabolites should be interpreted as predictive markers measured before or shortly after SVR rather than biomarkers measured at HCC onset. Metabolite levels may have changed during the interval between SVR confirmation and HCC diagnosis, and future serial metabolite measurements may further improve post-SVR HCC risk assessment.

Comparative analyses revealed that Met, MetO, Orn, and ACar exhibited significant Pre- to Post-Tx changes in the SVR group but not in the non-SVR group (Figure 2a). However, a subset of SVR patients appeared to show limited metabolic recovery after HCV clearance, raising the possibility that persistent metabolic alterations may be associated with ongoing liver disease progression and subsequent HCC risk. To investigate this possibility, we next examined the association between individual metabolites and post-SVR HCC development. Further analyses demonstrated that Pre-Tx Met, Pre- and Post-Tx Orn, and Post-Tx MetO were significantly associated with post-SVR HCC development, supporting their potential utility as predictive markers for risk stratification (Table 1 and Figure 4 and Figure 5).

To address the limited number of post-SVR HCC cases, we employed a two-step comparison strategy. First, comprehensive metabolomic analysis comparing SVR and non-SVR cases was used to identify treatment-responsive candidate biomarkers. Second, these candidates were evaluated in a comparison between post-SVR HCC and non-HCC cases to assess their utility for predicting post-SVR HCC risk. Although previous studies have reported metabolite-based predictors of HCC development in hepatitis C patients through comparisons between HCC and non-HCC cases [27,28,29,30], they did not specifically focus on post-SVR HCC. The present study identified candidate predictors of post-SVR HCC through comprehensive metabolomic profiling followed by a stepwise investigation of their clinical utility.

MetO and the MetO/Met ratio (MSR) have been used as indicators of oxidative stress in vivo [31,32]. In the present study, the post-treatment decrease in MSR in the SVR group may reflect attenuation of oxidative stress after viral clearance. Given that elevated Met and MetO have been associated with HCC risk in non-HCV-related cirrhosis [33,34], the increased Pre-Tx Met and Post-Tx MetO levels observed here support their potential utility as predictive biomarkers for post-SVR HCC. Methionine serves not only as the precursor of MetO but also as a central metabolite in pathways linked to glutathione synthesis and redox homeostasis. Therefore, alterations in Met and MetO may reflect broader metabolic changes associated with oxidative stress.

Metabolite levels may also be influenced by hepatic reserve, fibrosis, metabolic comorbidities (including diabetes and obesity), nutritional status, renal function, medications, alcohol intake, and systemic inflammation. Several of these factors were partially addressed in the present study through age matching, fasting sample collection, an assessment of body mass index and alcohol intake, the incorporation of fibrosis-related markers into the prediction models, and subgroup analyses excluding patients with diabetes (Supplementary Figure S7). Nevertheless, residual confounding from unmeasured factors, including renal function, sarcopenia, medication use, and other systemic conditions, cannot be excluded.

Notably, Met levels increased after SVR only in patients who did not develop HCC, whereas no such change was observed in those who later developed HCC. This pattern suggests that Met metabolism remained reversible after viral clearance in non-HCC patients, whereas less complete metabolic recovery may have occurred despite SVR in patients who later developed HCC. Thus, the significance of Met in this setting may lie less in its absolute level after treatment than in the presence or absence of metabolic recovery following SVR.

Orn is positioned at the intersection of arginine metabolism, the urea cycle, and polyamine synthesis. Polyamines are essential for cell proliferation and have long been implicated in hepatocarcinogenesis. Persistent elevation of Orn after SVR may therefore reflect sustained dysregulation of nitrogen metabolism and proliferative pathways. Nevertheless, Orn levels may also reflect broader hepatic and systemic metabolic conditions, and the present data do not establish a direct mechanistic role of Orn in post-SVR hepatocarcinogenesis.

The etiology of HCC after SVR appears to be multifactorial, limiting risk stratification based on a single biomarker. Although combinations of clinical and biochemical factors are generally considered to provide more accurate prognostic information than single serum markers in liver disease, only one predictive model incorporating FIB-4 index has been reported to date [24]. In the present study, combinations of FIB-4 with Met or Orn appeared to provide additional risk stratification compared with FIB-4 alone. In particular, the FIB-4 and Orn model identified a low-risk group with a 3-year HCC incidence of 0%, while the FIB-4 and Met model identified a low-risk group with a 3-year incidence of 6.4%.

Given the potential influence of diabetes and prior HCC on amino acid metabolism, we performed additional analyses excluding such patients. The results were largely unchanged and showed trends similar to those of the main analysis, suggesting an additive value of metabolites beyond FIB-4 alone (Supplementary Figure S7). Fibrosis severity matching was not performed, and residual confounding by fibrosis severity cannot be completely excluded. Nevertheless, this study was designed to identify metabolite markers that provide information beyond fibrosis-based risk assessment rather than merely reflect fibrosis severity. Importantly, the identified metabolites retained additive value in combination with fibrosis markers.

In the Post-Tx setting, FIB-4 index, AFP, MetO, and Orn were independently associated with post-SVR HCC development. Models incorporating MetO and Orn together with either FIB-4 index or AFP demonstrated acceptable discrimination after internal validation, with the AFP, MetO, and Orn model showing the highest optimism-corrected C-index among the evaluated models (Supplementary Table S10). In addition, these metabolite-based models showed higher optimism-corrected C-index values and provided better risk stratification than models based on FIB-4 index or AFP alone. Furthermore, no consensus has been reached regarding whether Pre- or Post-Tx factors are more useful for predicting HCC after SVR [35]. The present findings suggest that these two phases may provide complementary information, and future validation studies based on this framework may help clarify their relative clinical utility.

The absence of a healthy control group represents another limitation of the present study. As a result, it remains unclear whether the observed post-SVR metabolomic profiles reflect normalization, partial normalization, or persistent metabolic abnormalities compared with individuals without chronic liver disease. Although only one patient in the non-HCC group died before developing HCC during follow-up, competing risks were not formally considered and therefore may have influenced the estimated cumulative incidence of HCC.

In this study, 29 post-SVR HCC cases were analyzed. While this number is comparable to those in previous metabolomic studies of this type, the limited number of events may have affected the stability of the estimated cutoff values and hazard ratios, and the possibility of model overfitting cannot be completely excluded. Further prospective validation in larger independent cohorts is warranted, including patients with HCV serogroups other than serogroup 1 and those from non-Japanese ethnic backgrounds. Bootstrap optimism correction and calibration analyses supported the overall performance of the prediction models; however, external validation will be required to confirm their generalizability and clinical utility.

## 5. Conclusions

Serum Met, MetO, and Orn were identified as candidate metabolites associated with post-SVR HCC development in patients with HCV who achieved SVR. Both pre-treatment and post-treatment metabolite measurements may provide complementary information for risk stratification when combined with established clinical markers such as FIB-4 index and AFP. These findings suggest the potential utility of metabolite-based risk stratification for post-SVR HCC; however, further prospective validation in independent cohorts will be required to confirm their generalizability and clinical utility before clinical implementation.

## Figures and Tables

**Table 1:** Univariate analysis associated with HCC development after achieving SVR.

Factors	Pre-Tx			Post-Tx		
	HR	95% CI	*p*-Value	HR	95% CI	*p*-Value
***Liver function tests***						
AFP (ng/mL)	1.010	0.999–1.022	n.s.	1.193	1.113–1.280	<0.001
FIB-4 index	1.141	1.043–1.248	0.004	1.285	1.064–1.551	0.009
***Metabolites***						
Methionine (μg/mL)	1.347	1.169–1.551	<0.001	1.081	0.906–1.289	n.s.
Met-sulfoxide (×10^2^ ng/mL)	0.990	0.916–1.070	n.s.	1.115	1.018–1.222	0.020
Ornithine (μg/mL)	1.054	1.005–1.105	0.032	1.051	1.015–1.088	0.005
Acetyl-carnitine (μg/mL)	1.158	1.009–1.329	0.037	1.143	1.024–1.276	0.017
MSR	0.177	0.011–2.970	n.s.	2.497	0.530–11.77	n.s.

Hazard ratios were reported per 1 ng/mL increase for AFP, per 1-unit increase for FIB-4 index and MSR, per 1 µg/mL increase for Met, Orn, and ACar, and per 100 ng/mL increase for MetO. Variables were entered as continuous variables. Cox proportional hazard model; ns, not significant. Abbreviations: AFP, alpha-fetoprotein; CI, confidence interval; FIB-4, fibrosis-4; HCC, hepatocellular carcinoma; HR, hazard ratio; MSR, methionine–methionine sulfoxide ratio; n.s., not significant, Post-Tx, post-treatment; Pre-Tx, pre-treatment; SVR, sustained virological response.

**Table 2:** Multivariate analysis associated with development of HCC after achieving SVR.

**Pre-Tx**														
**Variables**	**Model 1**		**Model 2**		**Model 3**									
	**HR**	***p*** **-Value**	**HR**	***p*** **-Value**	**HR**	***p*** **-Value**								
	**(95% CI)**		**(95% CI)**		**(95% CI)**									
FIB-4 index	1.145 (1.038–1.263)	0.007	1.152 (1.052–1.261)	0.002	1.145 (1.037–1.265)	0.007								
Met (μg/mL)	1.353 (1.165–1.572)	<0.001	—		1.329 (1.135–1.558)	<0.001								
Orn (μg/mL)	-	-	1.063 (1.010–1.119)	0.02		n.s.								
**Post-Tx**														
**Variables**	**Model 1**		**Model 2**		**Model 3**		**Model 4**		**Model 5**		**Model 6**		**Model 7**	
	**HR**	***p*** **-Value**	**HR**	***p*** **-Value**	**HR**	***p*** **-Value**	**HR**	***p*** **-Value**	**HR**	***p*** **-Value**	**HR**	***p*** **-Value**	**HR**	***p*** **-Value**
	**(95% CI)**		**(95% CI)**		**(95% CI)**		**(95% CI)**		**(95% CI)**		**(95% CI)**		**(95% CI)**	
FIB-4 index	1.280 (1.054–1.554)	0.013	1.264 (1.044–1.530)	0.016	-	-	-	-	n.s.	n.s.	1.268 (1.039–1.548)	0.019	-	-
AFP (ng/mL)	-	-	-	-	1.193 (1.110–1.281)	<0.001	1.196 (1.114–1.284)	<0.001	3.598 (1.725–9.080)	0.001	-	-	1.191 (1.106–1.283)	<0.001
MetO (x10^2^ng/mL)	1.112 (1.012–1.220)	0.026	-	-	1.112 (1.005–1.231)	0.039	-	-	-	-	1.154 (1.044–1.276)	0.005	1.137 (1.022–1.266)	0.019
Orn (μg/mL)	-	-	1.05 (1.012–1.090)	0.01	-	-	1.047 (1.013–1.082)	0.006	-	-	1.059 (1.022–1.097)	0.001	1.05 (1.017–1.084)	0.003

The upper panel presents the results of multivariate analysis for HCC risk prediction models using Pre-Tx factors, showing Model 1 (FIB-4 index and Met), Model 2 (FIB-4 index and Orn), and Model 3 (FIB-4 index, Met, and Orn). The lower panel details Post-Tx factor analysis, including Model 1 (FIB-4 index and MetO), Model 2 (FIB-4 index and Orn), Model 3 (AFP and MetO), Model 4 (AFP and Orn), Model 5 (FIB-4 index and AFP), Model 6 (FIB-4 index, MetO, and Orn), and Model 7 (AFP, MetO, and Orn). Cox proportional hazard model; ns, not significant. Abbreviations: AFP, alpha-fetoprotein; CI, confidence interval; FIB-4, fibrosis-4; HCC, hepatocellular carcinoma; HR, hazard ratio; Met, methionine; MetO, methionine sulfoxide; n.s., not significant; Orn, ornithine; Post-Tx, post-treatment; Pre-Tx, pre-treatment; SVR, sustained virological response.

## Data Availability

The datasets generated and/or analyzed during this study are available upon reasonable request from the corresponding author.

## Supplementary Materials

The following supporting information can be downloaded at: https://www.mdpi.com/article/10.3390/cancers18122003/s1, Supporting Material and Methods [36]; Supplementary Figure S1: The LC-MS/MS spectrum of targeted metabolites and internal standards. The mass spectrum is represented with the horizontal axis (x-axis) showing the mass-to-charge ratio (m/z) and the vertical axis (y-axis) indicating the signal intensity, which corresponds to the abundance of detected ions. Four deuterium-labeled compounds were used as internal standards; methionine-d (MetD) for methionine (Met) and methionine sulfoxide (MetO), hydroxyproline-d (HypD) for hydroxyproline (Hyp), carnitine-d (CarD) for carnitine (Car), acetylcarnitine (ACar), asymmetric dimethylarginine (ADMA), and arginine (Arg), and ornithine-d (OrnD) for ornithine (Orn). Abbreviations: ACar, acetyl-carnitine; ADMA, asymmetric dimethylarginine; Arg, arginine; Car, carnitine; CarD, carnitine-d; Hyp, hydroxyproline; HypD, hydroxyproline-d; LC-MS/MS, liquid chromatography tandem mass spectrometry; Met, methionine; MetD, methionine-d; MetO, methionine sulfoxide; m/z, mass-to-charge ratio; Orn, ornithine; OrnD, ornithine-d; Supplementary Figure S2: The Euclidean distances obtained from Principal Component Analysis (PCA). The Euclidean distances in the PCA analyses were calculated for Exp 1 (a) and Exp 2 (b). Dots represent individual data, while bars represent the mean ± SD. * *p* < 0.05. Abbreviations: PCA, principal component analysis; Supplementary Figure S3: Loading scatter plot of the PCA analysis. Dots represent each metabolite. The horizontal axis represents PC1, and the vertical axis represents PC2. Abbreviations: ACar, acetyl-carnitine; ADMA, asymmetric dimethylarginine; Arg, arginine; Car, carnitine; Hyp, hydroxyproline; Met, methionine; MetO, methionine sulfoxide; Orn, ornithine; PC, principal component; Supplementary Figure S4: Calibration plots comparing predicted and observed 3-year HCC risk for the principal multivariable Cox models. Calibration was assessed using bootstrap resampling (B = 1000). The calibration time point was set at 3 years after SVR because relatively few patients were followed for 5 years. The dashed line represents ideal agreement between predicted and observed risk, and the solid line represents the optimism-corrected calibration curve. Abbreviations: HCC, hepatocellular carcinoma; Supplementary Figure S5: Receiver operating characteristic (ROC) curves for prediction of HCC development after achieving SVR. The horizontal axis represents 100 (%) − specificity (%), indicating the false positive rate. The vertical axis represents sensitivity (%), indicating the true positive rate. The optimal cut-off point of each factor for HCC prediction was defined as the point where the average of sensitivity and specificity reached its maximum (indicated by a dot in each figure). The cut-off points and their respective sensitivity and specificity for each factor are as follows: Pre-Tx FIB-4 index, cut-off point = 5.027, sensitivity = 72.4%, specificity = 67.2%. Pre-Tx Met, cut-off point = 3.708, sensitivity = 72.4%, specificity = 89.7%. Pre-Tx Orn, cut-off point = 18.863, sensitivity = 75.9%, specificity = 53.4%. Post-Tx FIB-4 index, cut-off point = 2.869, sensitivity = 86.2%, specificity = 44.8%. Post-Tx AFP, cut-off point = 5.300, sensitivity = 69.0%, specificity = 77.6%. Post-Tx MetO, cut-off point = 3.242, sensitivity = 44.8%, specificity = 91.4%. Post-Tx Orn, cut-off point = 17.067, sensitivity = 75.9%, specificity = 62.1%. These cutoff values were derived from the same cohort used for the subsequent stratification analyses; therefore, the estimates should be interpreted as exploratory. The area under the curve (AUC) value is calculated using the trapezoidal rule, which approximates the area under the ROC curve by dividing it into trapezoids between data points. The areas of these trapezoids are summed to get the total AUC, reflecting the discriminatory performance of the model. The corresponding AUC values are shown in each ROC plot. Abbreviations: AFP, alpha-fetoprotein; AUC, area under the curve; FIB-4, fibrosis 4; HCC, hepatocellular carcinoma; Met, methionine; MetO, methionine sulfoxide; Orn, ornithine; Post-Tx, post-treatment; Pre-Tx, pre-treatment; ROC, receiver operating characteristic; SVR, sustained virological response; Supplementary Figure S6: Cumulative incidences of HCC in patients who achieved SVR stratified by independent risk factors: Post-Tx serum metabolite levels and liver function test results. The cumulative incidences of HCC development after achievement of SVR were calculated when patients were stratified by the combination of FIB-4 index ≥ 2.869, AFP levels ≥5.3 ng/mL, and serum MetO levels ≥3.242 x102 ng/mL and by the combination of FIB-4 index ≥ 2.869, AFP levels ≥ 5.3 ng/mL, and Orn levels ≥ 17.067 µg/mL. The incidences were significantly different, with the 3-year HCC incidence in the high-risk group at 57.2% and 53.1%, respectively. The red, green and black lines represent the high-risk group (positive for two or three factors), medium-risk group (positive for one factor), and low-risk group (neither), respectively. The time (years) is measured from the point at which SVR was confirmed, specifically, 24 weeks after the end of treatment. Cumulative incidences were assessed using the Kaplan–Meier method and log-rank test. The ‘Number at risk’ indicates the number of subjects remaining at each time point who are still at risk of developing HCC. Log-rank test; * *p* < 0.05, ** *p* < 0.01, and *** *p* < 0.001. Abbreviations: AFP, alpha-fetoprotein; FIB-4, fibrosis 4; HCC, hepatocellular carcinoma; MetO, methionine sulfoxide; Orn, ornithine; SVR, sustained virological response; Supplementary Figure S7: Cumulative incidence of HCC in patients without a history of HCC or diabetes who achieved SVR. The cumulative incidence of HCC development after achievement of SVR in patients with neither prior HCC nor diabetes, stratified by independent risk factors (HCC group: *n* = 10, non-HCC group: *n* = 20). (a) Pre-Tx factors of FIB-4 index ≥ 5.027 and serum Met levels ≥ 3.708 µg/mL, and FIB-4 index ≥ 5.027 and serum Orn levels ≥ 18.863 µg/mL. (b) Post-Tx factors: FIB-4 index ≥ 2.869, serum MetO levels ≥3.242 × 10^2^ ng/mL, and Orn levels ≥ 17.067 µg/mL, and AFP levels ≥ 5.3 ng/mL, serum MetO levels ≥ 3.242 × 10^2^ ng/mL, and Orn levels ≥ 17.067 µg/mL. The red, green and black lines represent the high-risk group (positive for two or three factors), medium-risk group (positive for one factor), and low-risk group (neither). The time (years) is measured from the point at which SVR was confirmed, specifically, 24 weeks after the end of treatment. Cumulative incidence was evaluated using the Kaplan–Meier method and log-rank test. The ‘Number at risk’ indicates the number of subjects remaining at each time point who are still at risk of developing HCC. Log-rank test; * *p* < 0.05, ** *p* < 0.01, and *** *p* < 0.001. Abbreviations: AFP, alpha-fetoprotein; FIB-4, fibrosis 4; HCC, hepatocellular carcinoma; Met, methionine; MetO, methionine sulfoxide; Orn, ornithine; SVR, sustained virological response; Supplementary Table S1: Summary of Patients in the present study. Abbreviations: CE-TOFMS, capillary electrophoresis time-of-flight mass spectrometry; DAA, direct antiviral agent; Exp, experiment; HCC, hepatocellular carcinoma; LC-MS/MS, liquid chromatography tandem mass spectrometry; PegIFN, pegylated-interferon; PR, pegylated-interferon and ribavirin; Rbv, ribavirin; TPR, Telaprevir in combination with pegylated-interferon and ribavirin; SVR, sustained virological response; Supplementary Table S2: Characteristics of the enrolled patients for non-targeted metabolomics (Experiment 1). Serum samples were collected at four distinct time points shown in the figure, and the samples from each time point were designated as G1 through G4, respectively (G1: before the first treatment, G2: without viral disappearance after the first treatment, G3: before the second treatment, and G4: with viral clearance after the second treatment). Abbreviations: ALT, alanineaminotransferase; BMI, body mass index; DM, diabetes mellitus; FIB-4, fibrosis-4; HCC, hepatocellular carcinoma; HCV, hepatitis C virus; IFN, interferon; IL-28B, Interleukin-28B polymorphisms (rs8099917); PLT, platelet count; PR, pegylated-interferon and ribavirin; TPR, Telaprevir in combination with pegylated-interferon and ribavirin; SVR, sustained virological response; Supplementary Table S3: Characteristics of the enrolled patients for non-targeted metabolomics (Experiment 2). Serum samples were collected before treatment (TPR) and 24 weeks after treatment, and were designated as follows: G5 for pre-treatment, G6 for post-treatment with non-SVR, and G7 for post-treatment with SVR. Abbreviations: ALT, alanineaminotransferase; BMI, body mass index; DM, diabetes mellitus; FIB-4, fibrosis-4; HCC, hepatocellular carcinoma; HCV, hepatitis C virus; IFN, interferon; IL-28B, Interleukin-28B polymorphisms (rs8099917); ND, no data; PLT, platelet count; TPR, Telaprevir in combination with pegylated-interferon and ribavirin; SVR, sustained virological response; Supplementary Table S4: Measurement conditions for each compound in LC-MS/MS analysis. Abbreviations: ACar, acetyl-carnitine; ADMA, asymmetric dimethylarginine; Arg, arginine; Car, carnitine; CarD, carnitine-d; CE, Collision Energy; CXP, Collision Cell Exit Potential; DP, declustering potential; Hyp, hydroxyproline; HypD, hydroxyproline-d; LC-MS/MS, liquid chromatography tandem mass spectrometry; Met, methionine; MetD, methionine-d; MetO, methionine sulfoxide; Orn, ornithine; OrnD, ornithine-d; Q, Quadrupole; Table S5: Selection stability of candidate metabolites assessed by bootstrap resampling analyses. Candidate metabolites were initially selected based on their contribution to group separation in PCA loading scatter plots and their potential relevance to liver pathophysiology. Bootstrap resampling analyses (1000 resamples) were subsequently performed to evaluate the selection stability of these candidate metabolites. Values represent selection frequencies, defined as the proportion of bootstrap samples in which each metabolite was selected in the indicated group comparison. Higher values indicate greater selection stability across bootstrap resamples. Abbreviations: ACar, acetyl-carnitine; ADMA, asymmetric dimethylarginine; Arg, arginine; Car, carnitine; Exp, Experiment; G, group; Hyp, hydroxyproline; Met, methionine; MetO, methionine sulfoxide; Orn, ornithine; Supplementary Table S6: Characteristics of the enrolled patients with or without the achievement of SVR. Data are presented as mean (±SD) unless otherwise indicated. Mann–Whitney U test; ns, not significant. Abbreviations: ASV, asunaprevir; BMI, body mass index; DCV, daclatasvir; HCV, hepatitis C virus; GLE, glecaprevir; IFN, interferon; LDV, ledipasvir; PIB, pibrentasvir; Pre-Tx, pre-treatment; SOF, sofosbuvir; SVR, sustained virological response; Supplementary Table S7: Serum metabolites and liver function tests of patients with or without the achievement of SVR. The metabolite data are the same as that in Figure 2. Data are presented as mean (±SD). Wilcoxon signed-rank test; ns, not significant. Abbreviations: ADMA, asymmetric dimethylarginine; AFP, alpha-fetoprotein; ALBI, albumin–bilirubin; ALT, alanineaminotransferase; AST, aspartateaminotransferase; FIB-4, fibrosis-4; HCC, hepatocellular carcinoma; MSR, methionine–methionine sulfoxide ratio; OAR, ornithine–arginine ratio; Pre-Tx, pre-treatment; Post-Tx, post-treatment; SVR, sustained virological response; Supplementary Table S8: Characteristics of the enrolled patients with or without HCC development after achieving SVR. Data are presented as mean (±SD) unless otherwise indicated. Mann–Whitney U test; ns, not significant. Abbreviations: ASV, asunaprevir; BMI, body mass index; DCV, daclatasvir; HCV, hepatitis C virus; HCC, hepatocellular carcinoma; GLE, glecaprevir; IFN, interferon; LDV, ledipasvir; PIB, pibrentasvir; Pre-Tx, pre-treatment; SOF, sofosbuvir; Supplementary Table S9: Serum metabolites and liver function tests of patients with or without HCC development after achieving SVR. The metabolite data are the same as that in Figure 3. Data are presented as mean (±SD). Wilcoxon signed-rank test; ns, not significant. Abbreviations: AFP, alpha-fetoprotein; ALBI, albumin–bilirubin; ALT, alanineaminotransferase; AST, aspartateaminotransferase; FIB-4, fibrosis-4; HCC, hepatocellular carcinoma; MSR, methionine–methionine sulfoxide ratio; Pre-Tx, pre-treatment; Post-Tx, post-treatment; SVR, sustained virological response; Table S10: Apparent and optimism-corrected C-index values for clinically established and metabolite-based Cox prediction models. The apparent C-index represents the discrimination performance estimated in the original study cohort. Optimism-corrected C-index values were obtained using bootstrap resampling (B = 1000) to adjust for model optimism and provide an internally validated estimate of model discrimination. Abbreviations: AFP, alpha-fetoprotein; FIB-4, fibrosis 4; Met, methionine; MetO, methionine sulfoxide; Orn, ornithine; Post-Tx, post-treatment; Pre-Tx, pre-treatment.

## Abbreviations

The following abbreviations are used in this manuscript:

ACar	acetyl-carnitine
ADMA	asymmetric dimethylarginine
AFP	alpha-fetoprotein
ALBI	albumin–bilirubin
ALT	alanine aminotransferase
Arg	arginine
AST	aspartate aminotransferase
Car	carnitine
CE-TOFMS	capillary electrophoresis time-of-flight mass spectrometry
DAAs	direct-acting antivirals
Exp	experiment
FIB-4	fibrosis-4
HCA	hierarchical cluster analysis
HCC	hepatocellular carcinoma
HCV	hepatitis C virus
Hyp	hydroxyproline
IFN	interferon
LC-MS/MS	liquid chromatography tandem mass spectrometry
Met	methionine
MetO	methionine sulfoxide
MSR	methionine-methionine sulfoxide ratio
n.s.	not significant
OAR	ornithine-arginine ratio
Orn	ornithine
PCA	principal component analysis
PLS-DA	partial least squares discriminant analysis
Post-Tx	post-treatment
PR	pegylated interferon-α plus ribavirin
Pre-Tx	pre-treatment
ROC	receiver-operating characteristic
SVR	sustained virological response
TPR	Telaprevir plus PR

## References

[1] Bray F., Laversanne M., Sung H., Ferlay J., Siegel R.L., Soerjomataram I., Jemal A. (2024). Global cancer statistics 2022: GLOBOCAN estimates of incidence and mortality worldwide for 36 cancers in 185 countries. CA Cancer J. Clin..

[2] European Association for the Study of the Liver (2025). EASL Clinical Practice Guidelines on the management of hepatocellular carcinoma. J. Hepatol..

[3] Kanwal F., Kramer J., Asch S.M., Chayanupatkul M., Cao Y., El-Serag H.B. (2017). Risk of Hepatocellular Cancer in HCV Patients Treated with Direct-Acting Antiviral Agents. Gastroenterology.

[4] Waziry R., Hajarizadeh B., Grebely J., Amin J., Law M., Danta M., George J., Dore G.J. (2017). Hepatocellular carcinoma risk following direct-acting antiviral HCV therapy: A systematic review, meta-analyses, and meta-regression. J. Hepatol..

[5] D’ambrosio R., Ioannou G.N. (2021). Hepatocellular Carcinoma Risk, Outcomes, and Screening After Hepatitis C Eradication. Hepatol. Commun..

[6] Ahumada A., Rayón L., Usón C., Bañares R., Lopez S.A. (2021). Hepatocellular carcinoma risk after viral response in hepatitis C virus-advanced fibrosis: Who to screen and for how long?. World J. Gastroenterol..

[7] Ideno N., Nozaki A., Chuma M., Ogushi K., Hara K., Moriya S., Fukuda H., Numata K., Maeda S. (2022). Fib-4 index predicts prognosis after achievement of sustained virologic response following direct-acting antiviral treatment in patients with hepatitis C virus infection. Eur. J. Gastroenterol. Hepatol..

[8] Sou F.-M., Wu C.-K., Chang K.-C., Lu S.-N., Wang J.-H., Hung C.-H., Chen C.-H., Kee K.-M., Yen Y.-H., Lin M.-T. (2019). Clinical characteristics and prognosis of HCC occurrence after antiviral therapy for HCV patients between sustained and non-sustained responders. J. Formos. Med. Assoc..

[9] Tamaki N., Kurosaki M., Yasui Y., Mori N., Tsuji K., Hasebe C., Joko K., Akahane T., Furuta K., Kobashi H. (2021). Change in Fibrosis 4 Index as Predictor of High Risk of Incident Hepatocellular Carcinoma After Eradication of Hepatitis C Virus. Clin. Infect. Dis. Off. Publ. Infect. Dis. Soc. Am..

[10] Tanaka Y., Ogawa E., Huang C.-F., Toyoda H., Jun D.W., Tseng C.-H., Hsu Y.-C., Enomoto M., Takahashi H., For the REAL-C Investigators (2020). HCC risk post-SVR with DAAs in East Asians: Findings from the REAL-C cohort. Hepatol. Int..

[11] Pons M., Rodríguez-Tajes S., Esteban J.I., Mariño Z., Vargas V., Lens S., Buti M., Augustin S., Forns X., Mínguez B. (2020). Non-invasive prediction of liver-related events in patients with HCV-associated compensated advanced chronic liver disease after oral antivirals. J. Hepatol..

[12] Kumada T., Toyoda H., Yasuda S., Ogawa S., Gotoh T., Tada T., Ito T., Sumida Y., Tanaka J. (2022). Combined ultrasound and magnetic resonance elastography predict hepatocellular carcinoma after hepatitis C virus eradication. Hepatol. Res..

[13] Toyoda H., Tada T., Uojima H., Nozaki A., Chuma M., Takaguchi K., Hiraoka A., Abe H., Itobayashi E., Matsuura K. (2024). Comparison of six hepatocellular carcinoma prediction models in Japanese patients after sustained virologic response undergoing rigorous surveillance for hepatocellular carcinoma. J. Gastroenterol. Hepatol..

[14] Leslie J., Geh D., Elsharkawy A.M., Mann D.A., Vacca M. (2022). Metabolic dysfunction and cancer in HCV: Shared pathways and mutual interactions. J. Hepatol..

[15] Hamdane N., Jühling F., Crouchet E., El Saghire H., Thumann C., Oudot M.A., Bandiera S., Saviano A., Ponsolles C., Roca Suarez A.A.R. (2019). HCV-Induced Epigenetic Changes Associated with Liver Cancer Risk Persist after Sustained Virologic Response. Gastroenterology.

[16] Mamas M.B., Dunn W., Neyses L., Goodacre R. (2011). The role of metabolites and metabolomics in clinically applicable biomarkers of disease. Arch. Toxicol..

[17] Xia J., Broadhurst D.I., Wilson M., Wishart D.S. (2012). Translational biomarker discovery in clinical metabolomics: An introductory tutorial. Metabolomics.

[18] Zhou L., Ding L., Yin P., Lu X., Wang X., Niu J., Gao P., Xu G. (2012). Serum Metabolic Profiling Study of Hepatocellular Carcinoma Infected with Hepatitis B or Hepatitis C Virus by Using Liquid Chromatography–Mass Spectrometry. J. Proteome Res..

[19] Zeng J., Yin P., Tan Y., Dong L., Hu C., Huang Q., Lu X., Wang H., Xu G. (2014). Metabolomics Study of Hepatocellular Carcinoma: Discovery and Validation of Serum Potential Biomarkers by Using Capillary Electrophoresis–Mass Spectrometry. J. Proteome Res..

[20] Sato Y., Yamaguchi M., Kashiwakura I. (2022). An Analysis of the Serum Metabolomic Profile for the Radiomitigative Effect of the Thrombopoietin Receptor Agonist Romiplostim in Lethally Whole-Body-Irradiated Mice. Metabolites.

[21] Pang Z., Lu Y., Zhou G., Hui F., Xu L., Viau C., Spigelman A.F., MacDonald P.E., Wishart D.S., Li S. (2024). MetaboAnalyst 6.0: Towards a unified platform for metabolomics data processing, analysis and interpretation. Nucleic Acids Res..

[22] Ohashi Y., Hirayama A., Ishikawa T., Nakamura S., Shimizu K., Ueno Y., Tomita M., Soga T. (2007). Depiction of metabolome changes in histidine-starved *Escherichia coli* by CE-TOFMS. Mol. Biosyst..

[23] Rocha C., Doyle E.H., Bowman C.A., Fiel M., Stueck A.E., Goossens N., Bichoupan K., Patel N., Crismale J.F., Makkar J. (2023). Hepatocellular carcinoma in patients cured of chronic hepatitis C: Minimal steatosis. Cancer Med..

[24] Watanabe T., Tokumoto Y., Joko K., Michitaka K., Horiike N., Tanaka Y., Tada F., Kisaka Y., Nakanishi S., Yamauchi K. (2018). Predictors of hepatocellular carcinoma occurrence after direct-acting antiviral therapy in patients with hepatitis C virus infection. Hepatol. Res..

[25] Fitian A.I., Nelson D.R., Liu C., Xu Y., Ararat M., Cabrera R. (2014). Integrated metabolomic profiling of hepatocellular carcinoma in hepatitis C cirrhosis through GC/MS and UPLC/MS-MS. Liver Int..

[26] Beyoğlu D., Imbeaud S., Maurhofer O., Bioulac-Sage P., Zucman-Rossi J., Dufour J.-F., Idle J.R. (2013). Tissue metabolomics of hepatocellular carcinoma: Tumor energy metabolism and the role of transcriptomic classification. Hepatology.

[27] Patterson A.D., Maurhofer O., Beyoğlu D., Lanz C., Krausz K.W., Pabst T., Gonzalez F.J., Dufour J.-F., Idle J.R. (2011). Aberrant Lipid Metabolism in Hepatocellular Carcinoma Revealed by Plasma Metabolomics and Lipid Profiling. Cancer Res..

[28] Bowers J., Hughes E., Skill N., Maluccio M., Raftery D. (2014). Detection of hepatocellular carcinoma in hepatitis C patients: Biomarker discovery by LC–MS. J. Chromatogr. B.

[29] Lu Y., Li N., Gao L., Xu Y.-J., Huang C., Yu K., Ling Q., Cheng Q., Chen S., Zhu M. (2016). Acetylcarnitine Is a Candidate Diagnostic and Prognostic Biomarker of Hepatocellular Carcinoma. Cancer Res..

[30] Luo P., Yin P., Hua R., Tan Y., Li Z., Qiu G., Yin Z., Xie X., Wang X., Chen W. (2018). A Large-scale, multicenter serum metabolite biomarker identification study for the early detection of hepatocellular carcinoma. Hepatology.

[31] Berlett B.S., Stadtman E.R. (1997). Protein Oxidation in Aging, Disease, and Oxidative Stress. J. Biol. Chem..

[32] Chandran S., Binninger D. (2023). Role of Oxidative Stress, Methionine Oxidation and Methionine Sulfoxide Reductases (MSR) in Alzheimer’s Disease. Antioxidants.

[33] Dong T.S., Jacobs J.P., Agopian V., Pisegna J.R., Ayoub W., Durazo F., Enayati P., Sundaram V., Benhammou J.N., Noureddin M. (2021). Duodenal Microbiome and Serum Metabolites Predict Hepatocellular Carcinoma in a Multicenter Cohort of Patients with Cirrhosis. Dig. Dis. Sci..

[34] Morine Y., Utsunomiya T., Yamanaka-Okumura H., Saito Y., Yamada S., Ikemoto T., Imura S., Kinoshita S., Hirayama A., Tanaka Y. (2022). Essential amino acids as diagnostic biomarkers of hepatocellular carcinoma based on metabolic analysis. Oncotarget.

[35] Nakai M., Yamamoto Y., Baba M., Suda G., Kubo A., Tokuchi Y., Kitagataya T., Yamada R., Shigesawa T., Suzuki K. (2022). Prediction of hepatocellular carcinoma using age and liver stiffness on transient elastography after hepatitis C virus eradication. Sci. Rep..

[36] Caviglia G.P., Troshina G., Santaniello U., Rosati G., Bombaci F., Birolo G., Nicolosi A., Saracco G.M., Ciancio A. (2022). Long-Term Hepatocellular Carcinoma Development and Predictive Ability of Non-Invasive Scoring Systems in Patients with HCV-Related Cirrhosis Treated with Direct-Acting Antivirals. Cancers.

