# Supplementary material for: Metabolomics Approach Identifies Predictive Serum Markers for Hepatocellular Carcinogenesis Following Hepatitis C Virus Elimination
Source: Cancers (Basel). 2026 Jun 20;18(12):2003. doi: 10.3390/cancers18122003 (PMC13297395; doi:10.3390/cancers18122003)
Supplement: Supplementary file 1 [file cancers-18-02003-s001.zip › cancers-4317307-supplementary.pdf]

cancers

MDPI

Supplementary Materials

Supporting Material and Methods

Liver function test

Hematological and biochemical parameters were measured at each institution using standard techniques. Serum HCV-RNA titers were measured using the Roche COBAS TaqMan test (LLQ; 1.2 log10 IU/mL; Roche Molecular Diagnostics, CA) or AccuGene m-HCV (LLQ; 1.1 log10 IU/mL; Abbott Japan, Tokyo, Japan). The FIB-4 index and albumin-bilirubin (ALBI) scores were calculated and used as surrogate indicators of liver fibrosis and function, respectively [36].

LC-MS/MS with multiple reaction monitoring (MRM) technique for the measurement of individual candidate metabolites

Purified products of each metabolite were purchased and used as standard stocks and optically deuterated compounds were used as IS (See Supplementary Figure S1). Thirty microliter aliquots of serum samples were subjected to standard methanol extraction. The aqueous phase was filtered through a syringe-driven filter unit (PTFE, 0.2 µm pore size; Millipore, Billerica, MA, USA) and then analyzed using LC–MS/MS. Quality control samples were processed alongside to monitor the stability of the large-scale analysis. The LC system consisted of an Acquity ultra performance liquid chromatography (UPLC) unit (Waters, Milford, MA, USA). The samples were separated on a Discovery HS F5 HPLC Column (150 mm length × 2.1 mm i.d., particle size 3 µm; Sigma-Aldrich, St. Louis, MO, USA). The mobile phases were 5mM ammonium formate and 0.1% formic acid in water (A) and 0.1% formic acid in acetonitrile (ACN) (B), and the flow rate was 0.25 mL/min. After injection, mobile phase B was held at 0% for 2 minutes, then linearly increased to 25% over the next 3 minutes, and further increased to 35% over the following 6 minutes, followed by a rapid increase to 95% in 1 minute. The proportion of B was maintained at 95% for additional 3 minutes and then rapidly reduced to 0% in 0.1 minute for re-equilibration. The total run time for each sample was 25 minutes. The MS/MS analysis was performed in positive ESI mode using a 4000Q-TRAP quadropole linear ion-trap hybrid mass spectrometer (AB SCIEX, Framingham, MA, USA) with the following settings: ion spray needle voltage, 5500 V and ion source temperature, 700°C. The measurement conditions for each compound in tandem MS analysis with multiple reaction monitoring are shown in Supplementary Table S4. The representative peak profiles obtained in the chromatograms using each standard are shown in Supplementary Figure S1.

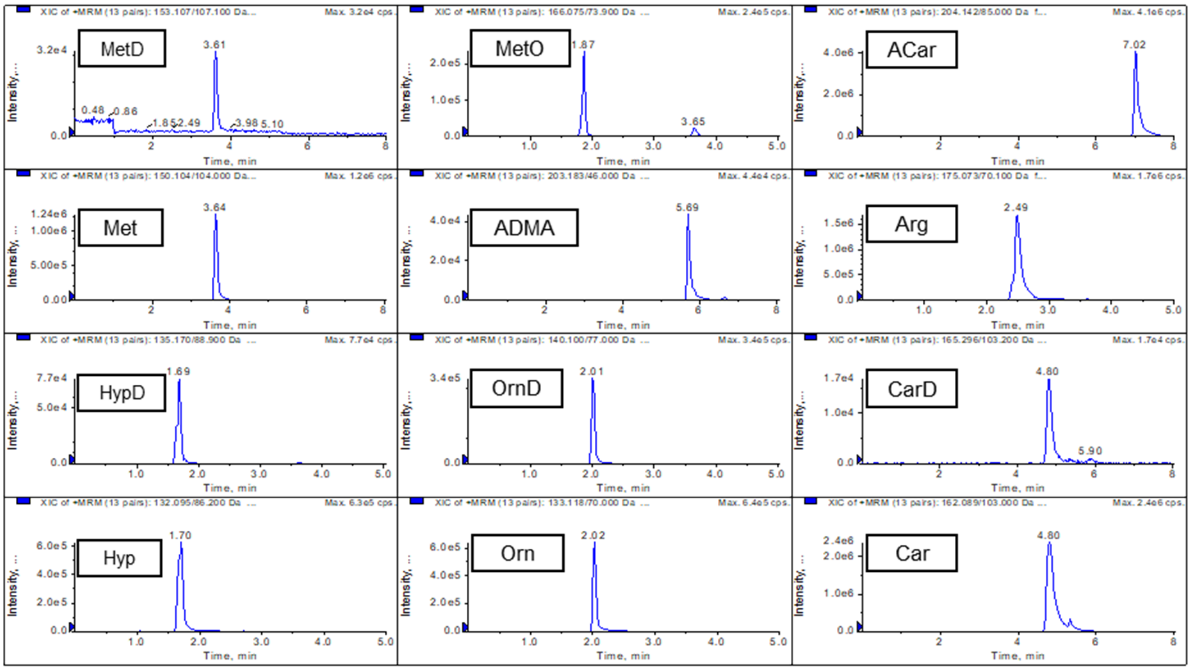

Figure S1. The LC-MS/MS spectrum of targeted metabolites and internal standards. The mass spectrum is represented with the horizontal axis (x-axis) showing the mass-to-charge ratio (m/z) and the vertical axis (y-axis) indicating the signal intensity, which corresponds to the abundance of detected ions. Four deuterium-labeled compounds were used as internal standards; methionine-d (MetD) for methionine (Met) and methionine sulfoxide (MetO), hydroxyproline-d (HypD) for hydroxyproline (Hyp), carnitine-d (CarD) for carnitine (Car), acetylcarnitine (ACar), asymmetric dimethylarginine (ADMA), and arginine (Arg), and ornithine-d (OrnD) for ornithine (Orn). Abbreviations: ACar, acetyl-carnitine; ADMA, asymmetric dimethylarginine; Arg, arginine; Car, carnitine; CarD, carnitine-d; Hyp, hydroxyproline; HypD, hydroxyproline-d; LC-MS/MS, liquid chromatography tandem mass spectrometry; Met, methionine; MetD, methionine-d; MetO, methionine sulfoxide; m/z, mass-to-charge ratio; Orn, ornithine; OrnD, ornithine-d.

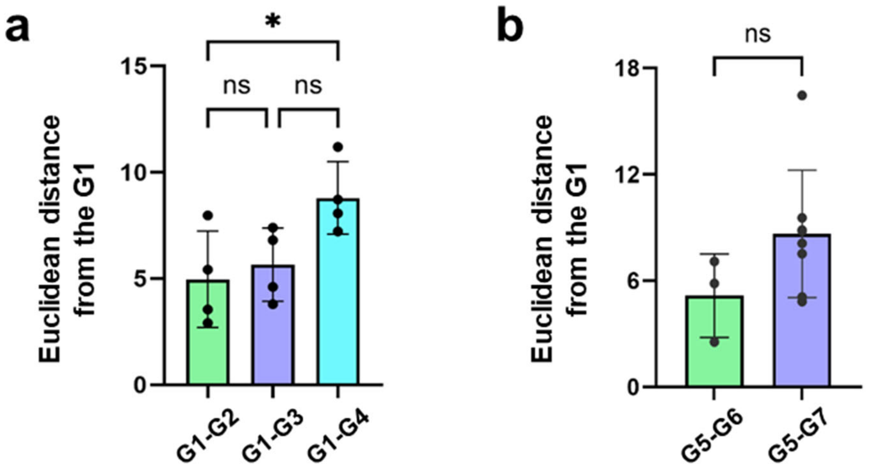

Figure S2. The Euclidean distances obtained from Principal Component Analysis (PCA). The Euclidean distances in the PCA analyses were calculated for Exp 1 (a) and Exp 2 (b). Dots represent individual data, while bars represent the mean ± SD. * $p < 0.05$. Abbreviations: PCA, principal component analysis.

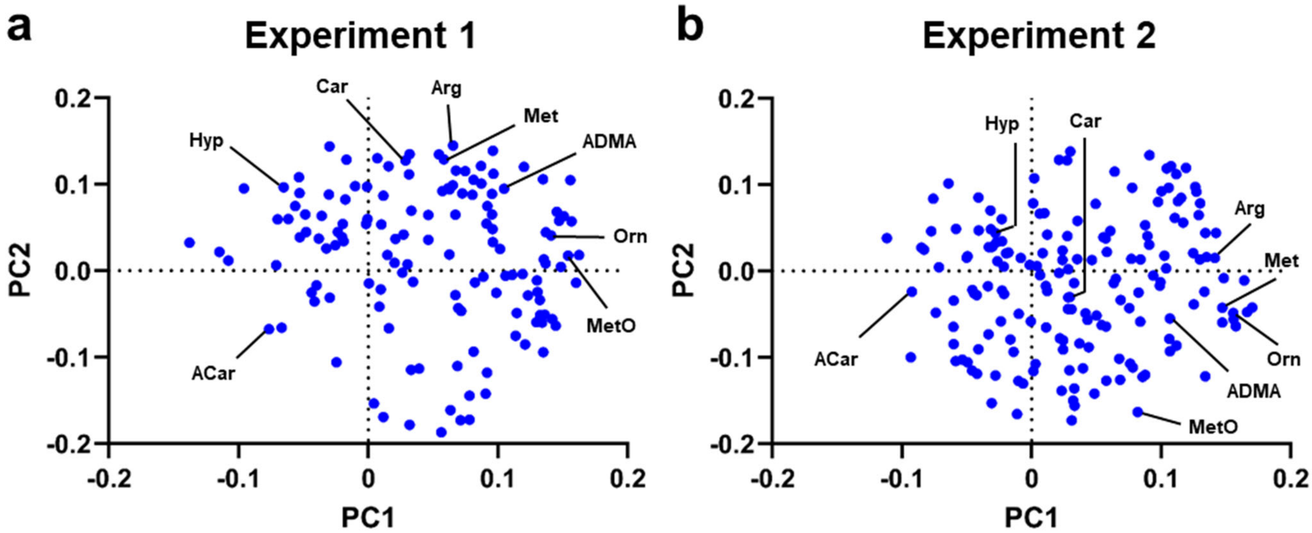

Figure S3. Loading scatter plot of the PCA analysis. Dots represent each metabolite. The horizontal axis represents PC1, and the vertical axis represents PC2. Abbreviations: ACar, acetyl-carnitine; ADMA, asymmetric dimethylarginine; Arg, arginine; Car, carnitine; Hyp, hydroxyproline; Met, methionine; MetO, methionine sulfoxide; Orn, ornithine; PC, principal component.

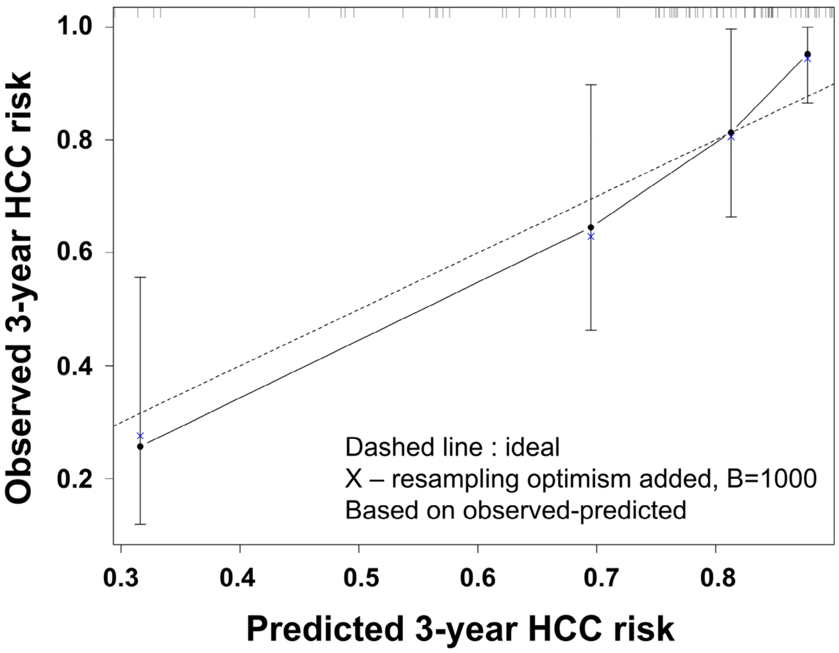

Figure S4. Calibration plots comparing predicted and observed 3-year HCC risk for the principal multivariable Cox models. Calibration was assessed using bootstrap resampling (B = 1000). The calibration time point was set at 3 years after SVR because relatively few patients were followed for 5 years. The dashed line represents ideal agreement between predicted and observed risk, and the solid line represents the optimism-corrected calibration curve. Abbreviations: HCC, hepatocellular carcinoma.

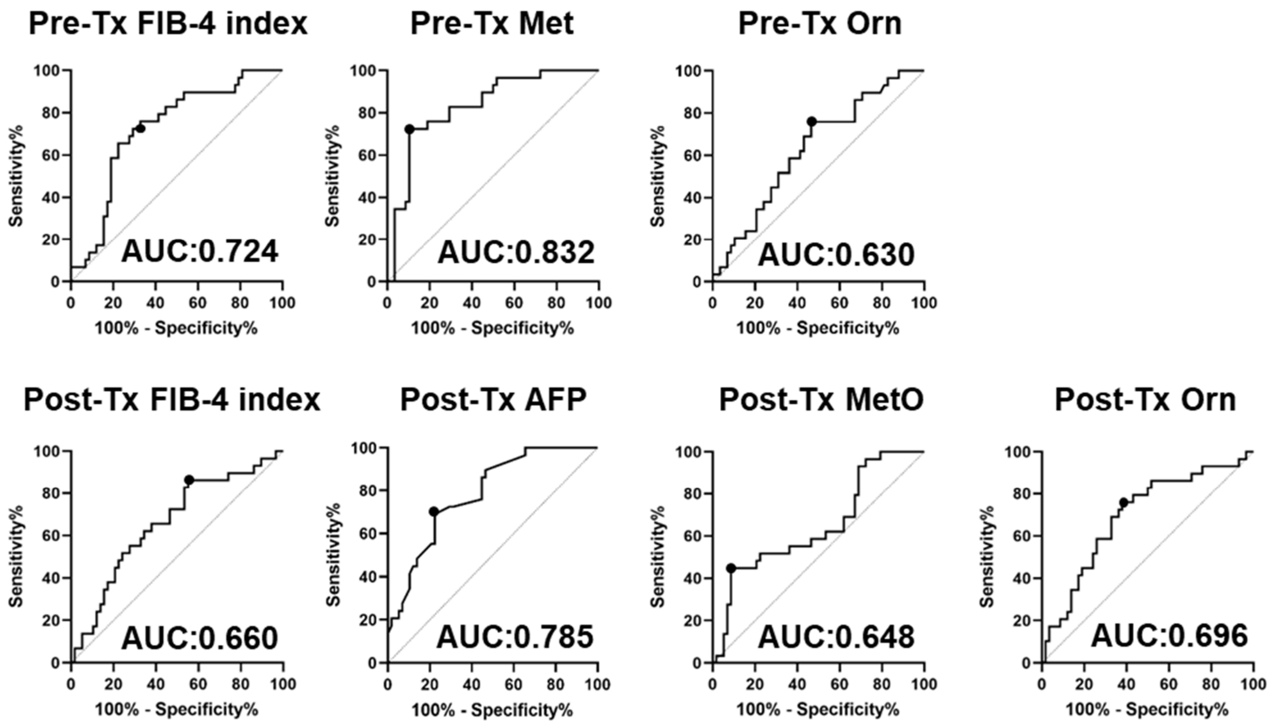

Figure S5. Receiver operating characteristic (ROC) curves for prediction of HCC development after achieving SVR. The horizontal axis represents 100 (%) − specificity (%), indicating the false positive rate. The vertical axis represents sensitivity (%), indicating the true positive rate. The optimal cut-off point of each factor for HCC prediction was defined as the point where the average of sensitivity and specificity reached its maximum (indicated by a dot in each figure). The cut-off points and their respective sensitivity and specificity for each factor are as follows: Pre-Tx FIB-4 index, cut-off point = 5.027, sensitivity = 72.4%, specificity = 67.2%. Pre-Tx Met, cut-off point = 3.708, sensitivity = 72.4%, specificity = 89.7%. Pre-Tx Orn, cut-off point = 18.863, sensitivity = 75.9%, specificity = 53.4%. Post-Tx FIB-4 index, cut-off point = 2.869, sensitivity = 86.2%, specificity = 44.8%. Post-Tx AFP, cut-off point = 5.300, sensitivity = 69.0%, specificity = 77.6%. Post-Tx MetO, cut-off point = 3.242, sensitivity = 44.8%, specificity = 91.4%. Post-Tx Orn, cut-off point = 17.067, sensitivity = 75.9%, specificity = 62.1%. These cutoff values were derived from the same cohort used for the subsequent stratification analyses; therefore, the estimates should be interpreted as exploratory. The area under the curve (AUC) value is calculated using the trapezoidal rule, which approximates the area under the ROC curve by dividing it into trapezoids between data points. The areas of these trapezoids are summed to get the total AUC, reflecting the discriminatory performance of the model. The corresponding AUC values are shown in each ROC plot. Abbreviations: AFP, alpha-fetoprotein; AUC, area under the curve; FIB-4, fibrosis 4; HCC, hepatocellular carcinoma; Met, methionine; MetO, methionine sulfoxide; Orn, ornithine; Post-Tx, post-treatment; Pre-Tx, pre-treatment; ROC, receiver operating characteristic; SVR, sustained virological response.

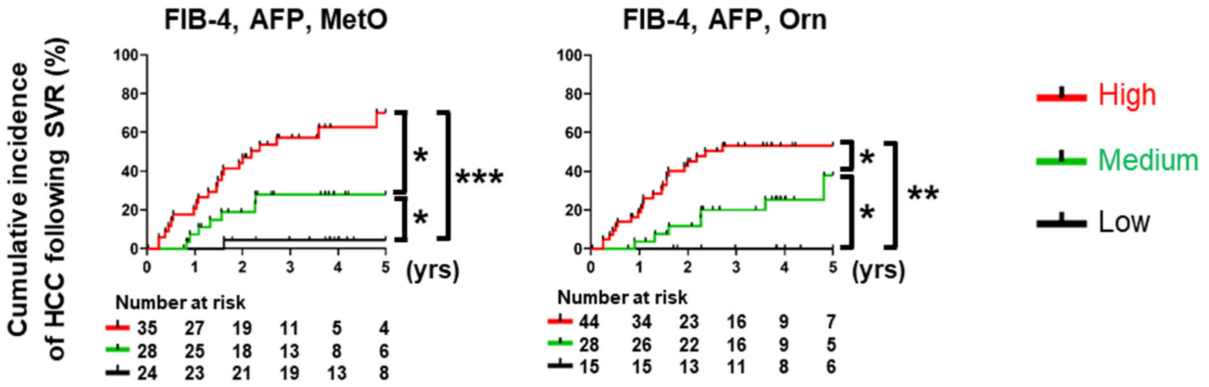

Figure S6. Cumulative incidences of HCC in patients who achieved SVR stratified by independent risk factors: Post-Tx serum metabolite levels and liver function test results. The cumulative incidences of HCC development after achievement of SVR were calculated when patients were stratified by the combination of FIB-4 index ≥ 2.869, AFP levels ≥5.3 ng/mL, and serum MetO levels ≥ 3.242 x102 ng/mL and by the combination of FIB-4 index ≥ 2.869, AFP levels ≥5.3 ng/mL, and Orn levels ≥ 17.067 µg/mL. The incidences were significantly different, with the 3-year HCC incidence in the high-risk group at 57.2% and 53.1%, respectively. The red, green and black lines represent the high-risk group (positive for two or three factors), medium-risk group (positive for one factor), and low-risk group (neither), respectively. The time (years) is measured from the point at which SVR was confirmed, specifically, 24 weeks after the end of treatment. Cumulative incidences were assessed using the Kaplan–Meier method and log-rank test. The 'Number at risk' indicates the number of subjects remaining at each time point who are still at risk of developing HCC. Log-rank test; * $p < 0.05$, ** $p < 0.01$, and *** $p < 0.001$. Abbreviations: AFP, alpha-fetoprotein; FIB-4, fibrosis 4; HCC, hepatocellular carcinoma; MetO, methionine sulfoxide; Orn, ornithine; SVR, sustained virological response.

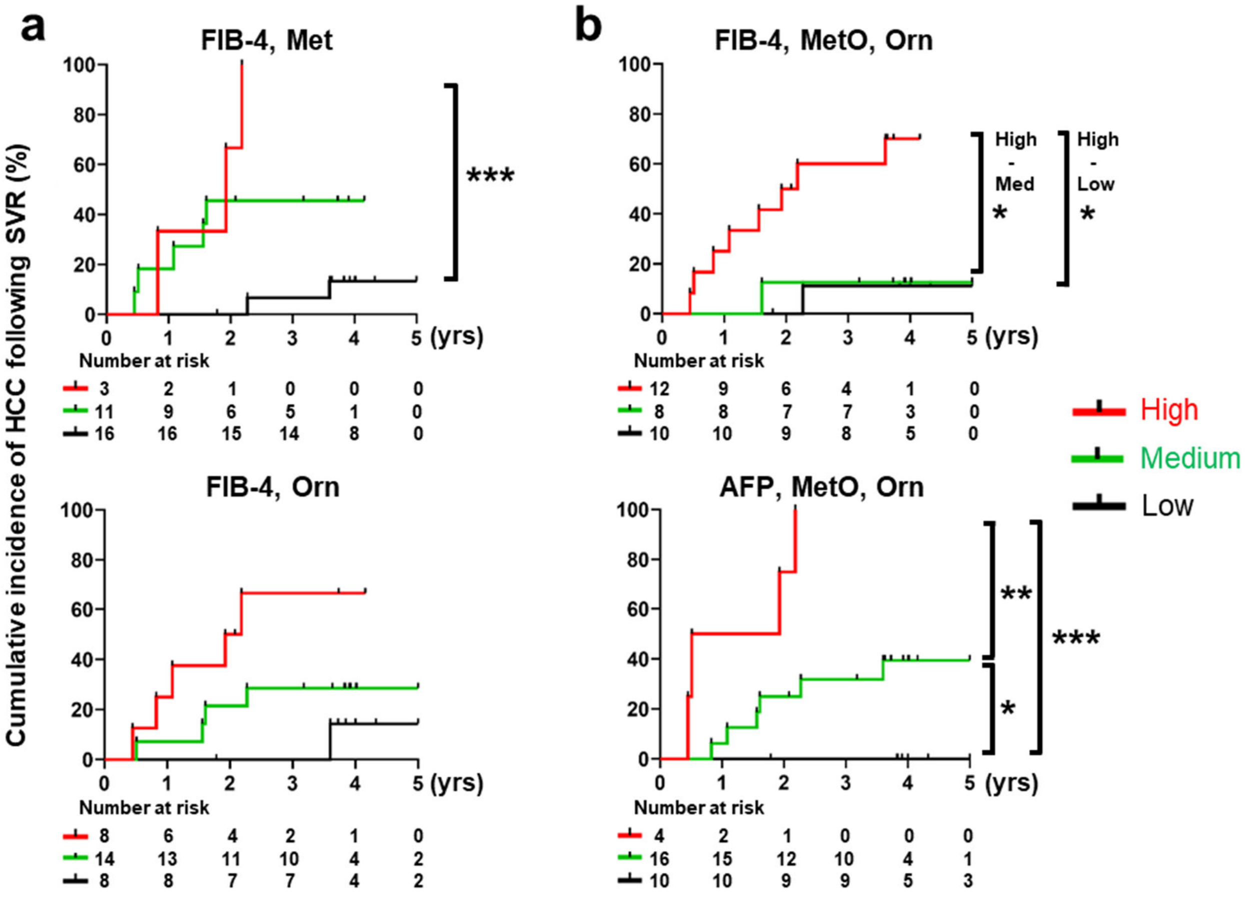

Figure S7. Cumulative incidence of HCC in patients without a history of HCC or diabetes who achieved SVR. The cumulative incidence of HCC development after achievement of SVR in patients with neither prior HCC nor diabetes, stratified by independent risk factors (HCC group: N = 10, non-HCC group: N = 20). (a) Pre-Tx factors of FIB-4 index ≥ 5.027 and serum Met levels ≥ 3.708 µg/mL, and FIB-4 index ≥ 5.027 and serum Orn levels ≥ 18.863 µg/mL. (b) Post-Tx factors: FIB-4 index ≥ 2.869, serum MetO levels ≥3.242 × 10^2 ng/mL, and Orn levels ≥ 17.067 µg/mL, and AFP levels ≥ 5.3 ng/mL, serum MetO levels ≥ 3.242 × 10^2 ng/mL, and Orn levels ≥ 17.067 µg/mL. The red, green and black lines represent the high-risk group (positive for two or three factors), medium-risk group (positive for one factor), and low-risk group (neither). The time (years) is measured from the point at which SVR was confirmed, specifically, 24 weeks after the end of treatment. Cumulative incidence was evaluated using the Kaplan–Meier method and log-rank test. The 'Number at risk' indicates the number of subjects remaining at each time point who are still at risk of developing HCC. Log-rank test; * $p < 0.05$, ** $p < 0.01$, and *** $p < 0.001$. Abbreviations: AFP, alpha-fetoprotein; FIB-4, fibrosis 4; HCC, hepatocellular carcinoma; Met, methionine; MetO, methionine sulfoxide; Orn, ornithine; SVR, sustained virological response.

Table S1. Summary of Patients in the present study. Abbreviations: CE-TOFMS, capillary electrophoresis time-of-flight mass spectrometry; DAA, direct antiviral agent; Exp, experiment; HCC, hepatocellular carcinoma; LC-MS/MS, liquid chromatography tandem mass spectrometry; PegIFN, pegylated-interferon; PR, pegylated-interferon and ribavirin; Rbv, ribavirin; TPR, Telaprevir in combination with pegylated-interferon and ribavirin; SVR, sustained virological response.

Exp	Therapy	Analysis	Patient groups	Patients background	Results
1	1st: PegIFNα / Rbv (PR)<br>2nd: Telaprevir / PegIFNα / Rbv (TPR)	CE-TOFMS<br>(Semi-quantitative)	non-SVR with PR followed by SVR with TPR<br>(N=4)	Suppl Table S2	Figure 1<br>Suppl Figure S2, S3
2	Telaprevir / PegIFNα / Rbv (TPR)	CE-TOFMS<br>(Semi-quantitative)	non-SVR (N=3)<br>SVR (N=8)	Suppl Table S3	Figure 1<br>Suppl Figure S2, S3
3	Interferon-free DAA	LC-MS/MS<br>(Absolute quantification)	non-SVR (N=17)<br>SVR (N=34)	Suppl Table S6	Figure 2<br>Suppl Table S7
4	Interferon-free DAA	LC-MS/MS<br>(Absolute quantification)	HCC (N=29)<br>non-HCC (N=58)	Suppl Table S8	Figures 3–5<br>Tables 1, 2<br>Suppl Figure S5, S6<br>Suppl Table S9

Table S2. Characteristics of the enrolled patients for non-targeted metabolomics (Experiment 1). Serum samples were collected at four distinct time points shown in the figure, and the samples from each time point were designated as G1 through G4, respectively (G1: before the first treatment, G2: without viral disappearance after the first treatment, G3: before the second treatment, and G4: with viral clearance after the second treatment). Abbreviations: ALT, alanineaminotransferase; BMI, body mass index; DM, diabetes mellitus; FIB-4, fibrosis-4; HCC, hepatocellular carcinoma; HCV, hepatitis C virus; IFN, interferon; IL-28B, Interleukin-28B polymorphisms (rs8099917); PLT, platelet count; PR, pegylated-interferon and ribavirin; TPR, Telaprevir in combination with pegylated-interferon and ribavirin; SVR, sustained virological response.

Case	Age yr	Sex	Sero group	BMI kg/m^2	IL-28B	DM	Prior IFN	Prior HCC	METAVIR score	PLT $x10^9/L$	ALT U/L	FIB-4 index
a	60	F	1	21.4	T/G	N	Y	N	A1F0	17.4	44	2.61
b	58	F	1	18.9	T/G	N	N	N	A2F2	19.3	62	2.05
c	55	F	1	17.8	T/T	N	N	N	A2F2	13.5	28	2.56
d	58	F	1	23.8	G/G	N	N	N	A2F3	13.7	122	4.41

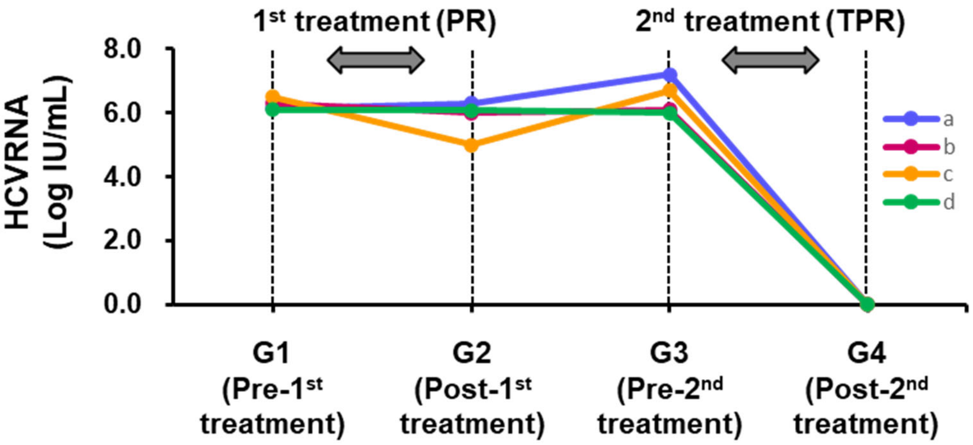

Table S3. Characteristics of the enrolled patients for non-targeted metabolomics (Experiment 2). Serum samples were collected before treatment (TPR) and 24 weeks after treatment, and were designated as follows: G5 for pre-treatment, G6 for post-treatment with non-SVR, and G7 for post-treatment with SVR. Abbreviations: ALT, alanineaminotransferase; BMI, body mass index; DM, diabetes mellitus; FIB-4, fibrosis-4; HCC, hepatocellular carcinoma; HCV, hepatitis C virus; IFN, interferon; IL-28B, Interleukin-28B polymorphisms (rs8099917); ND, no data; PLT, platelet count; TPR, Telaprevir in combination with pegylated-interferon and ribavirin; SVR, sustained virological response.

Case	Age yr	Sex	SVR	Sero group	BMI kg/m^2	IL-28B	DM	Prior IFN	Prior HCC	META-VIR score	PLT $x10^9/L$	ALT U/L	FIB-4 index	HCVRNA LogIU/mL
e	62	M	N	1	24.2	T/G	N	Y	N	A1F1	161	201	3.04	5.5
f	50	M	N	1	25.7	T/T	N	Y	N	A3F3	135	222	5.15	7.3
g	67	F	N	1	24.0	T/G	N	N	N	ND	240	55	2.18	6.9
h	56	M	Y	1	24.3	T/T	N	N	N	A2F2	104	320	5.72	6.6
i	66	M	Y	1	18.4	T/T	N	N	N	A2F1	143	73	2.97	6.9
j	68	F	Y	1	21.8	T/T	N	N	N	A1F1	155	40	2.29	7.1
k	63	F	Y	1	21.4	T/G	N	Y	N	A1F0	249	26	1.29	7.2
l	60	F	Y	1	18.9	T/G	N	Y	N	A2F2	208	68	1.75	6.1
m	65	F	Y	1	18.2	T/T	N	Y	N	A2F2	170	15	1.97	6.7
n	62	M	Y	1	22.2	T/T	N	N	N	A2F3	142	57	2.43	7.1
o	61	M	Y	1	19.4	T/T	N	N	N	A1F1	192	29	2.95	6.1

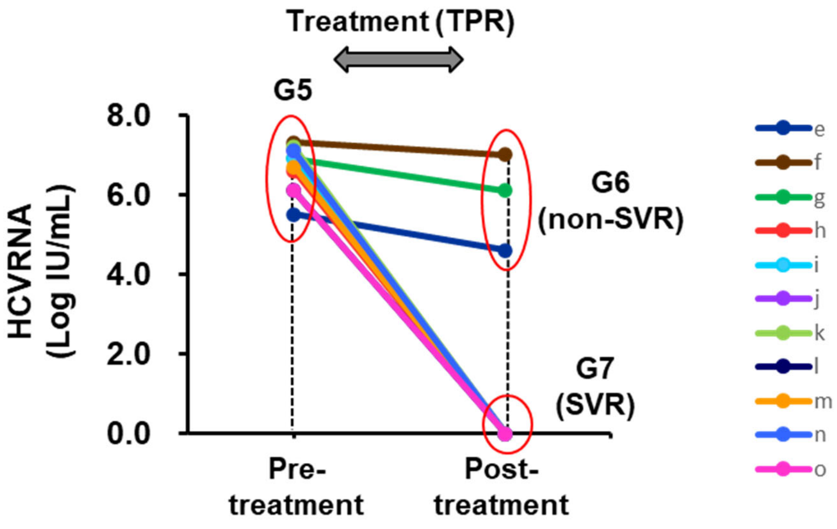

Table S4. Measurement conditions for each compound in LC-MS/MS analysis. Abbreviations: ACar, acetyl-carnitine; ADMA, asymmetric dimethylarginine; Arg, arginine; Car, carnitine; CarD, carnitine-d; CE, Collision Energy; CXP, Collision Cell Exit Potential; DP, declustering potential; Hyp, hydroxyproline; HypD, hydroxyproline-d; LC-MS/MS, liquid chromatography tandem mass spectrometry; Met, methionine; MetD, methionine-d; MetO, methionine sulfoxide; Orn, ornithine; OrnD, ornithine-d; Q, Quadrupole.

Metabolite	Q1/Q3 (Da)	DP (V)	CE (eV)	CXP (V)
Met	150.104 / 104.000	41	15	14
MetO	166.075 / 73.900	56	17	0
MetD	153.107 / 107.100	61	15	4
Hyp	132.095 / 86.200	56	21	4
HypD	135.170 / 88.900	120	21	12
Car	162.089 / 103.000	61	23	10
CarD	165.296 / 103.200	51	25	4
Orn	133.118 / 70.000	56	27	10
OrnD	140.100 / 77.000	71	27	10
ADMA	203.183 / 46.000	51	37	8
Arg	175.073 / 70.100	56	39	12
ACar	204.142 / 85.000	66	25	12

Table S5. Selection stability of candidate metabolites assessed by bootstrap resampling analyses. Candidate metabolites were initially selected based on their contribution to group separation in PCA loading scatter plots and their potential relevance to liver pathophysiology. Bootstrap resampling analyses (1000 resamples) were subsequently performed to evaluate the selection stability of these candidate metabolites. Values represent selection frequencies, defined as the proportion of bootstrap samples in which each metabolite was selected in the indicated group comparison. Higher values indicate greater selection stability across bootstrap resamples. Abbreviations: ACar, acetyl-carnitine; ADMA, asymmetric dimethylarginine; Arg, arginine; Car, carnitine; Exp, Experiment; G, group; Hyp, hydroxyproline; Met, methionine; MetO, methionine sulfoxide; Orn, ornithine.

**Exp 1**						
	**G1 vs G2**	**G1 vs G3**	**G1 vs G4**	**G2 vs G3**	**G2 vs G4**	**G3 vs G4**
Met	0.093	0.066	0.086	0.078	0.069	0.052
MetO	0.048	0.414	0.865	0.788	0.999	0.931
Hyp	0.094	0.08	0.583	0.095	0.778	0.495
Car	0.04	0.056	0.056	0.06	0.059	0.076
Orn	0.063	0.127	0.266	0.134	0.456	0.434
ADMA	0.077	0.095	0.062	0.345	0.107	0.122
Arg	0.108	0.105	0.075	0.341	0.837	0.096
ACar	0.044	0.109	0.117	0.072	0.065	0.062
**Exp 2**						
	**G1 vs G2**	**G1 vs G3**	**G2 vs G3**			
Met	0.248	0.082	0.313			
MetO	0.074	0.986	0.331			
Hyp	0.29	0.677	0.334			
Car	0.134	0.142	0.074			
Orn	0.095	0.212	0.081			
ADMA	0.294	0.263	0.096			
Arg	0.378	0.177	0.13			
ACar	0.09	0.272	0.314			

Table S6. Characteristics of the enrolled patients with or without the achievement of SVR. Data are presented as mean (±SD) unless otherwise indicated. Mann–Whitney U test; ns, not significant. Abbreviations: ASV, asunaprevir; BMI, body mass index; DCV, daclatasvir; HCV, hepatitis C virus; GLE, glecaprevir; IFN, interferon; LDV, ledipasvir; PIB, pibrentasvir; Pre-Tx, pre-treatment; SOF, sofosbuvir; SVR, sustained virological response.

Factors	non-SVR	SVR	*p*-value
Patients, n	17	34	
Age (yr)	70 ± 9	70 ± 9	n.s.
Sex (male, n (%))	9 (52.9)	17 (50.0)	n.s.
BMI (kg/m^2)	22.8 ± 3.6	21.6 ± 2.5	n.s.
Past history of IFN treatment, n (%)	9 (52.9)	12 (70.1)	n.s.
HCV serogroup1, n (%)	17 (100)	32 (94.1)	n.s.
Treatment, n (%)			
DCV/ASV	13 (76.5)	16 (47.1)	n.s.
SOF/LDV	3 (17.6)	15 (44.1)	n.s.
GLE/PIB	0 (0)	3 (8.8)	n.s.
Others	1 (5.9)	0 (0)	n.s.
Pre-Tx HCV titer (Log IU/mL)	6.1 ± 0.4	6.1 ± 0.5	n.s.

Table S7. Serum metabolites and liver function tests of patients with or without the achievement of SVR. The metabolite data are the same as that in Figure 2. Data are presented as mean (±SD). Wilcoxon signed-rank test; ns, not significant. Abbreviations: ADMA, asymmetric dimethylarginine; AFP, alpha-fetoprotein; ALBI, albumin–bilirubin; ALT, alanineaminotransferase; AST, aspartateam-inotransferase; FIB-4, fibrosis-4; HCC, hepatocellular carcinoma; MSR, methionine–methionine sulfoxide ratio; OAR, ornithine–arginine ratio; Pre-Tx, pre-treatment; Post-Tx, post-treatment; SVR, sustained virological response.

Factors	non-SVR			SVR		
	Pre-Tx	Post-Tx	*p*-value	Pre-Tx	Post-Tx	*p*-value
Liver function tests						
AST (U/L)	69 ± 45	56 ± 37	0.036	47 ± 26	24 ± 6	<0.001
ALT (U/L)	56 ± 30	65 ± 89	n.s.	38 ± 20	15 ± 7	<0.001
Platelet counts (×10^9/L)	127 ± 53	128 ± 5.0	n.s.	149 ± 54	159 ± 54	n.s.
T. Bilirubin (mg/dL)	0.87 ± 0.33	0.79 ± 0.26	n.s.	0.85 ± 0.32	0.86 ± 0.40	n.s.
Albumin (g/dL)	3.9 ± 0.4	3.9 ± 0.5	n.s.	4.0 ± 0.4	4.2 ± 0.4	0.002
AFP (ng/mL)	21.5 ± 28.8	10.3 ± 8.2	0.010	9.1 ± 9.1	4.4 ± 2.7	<0.001
ALBI score	-2.52 ± 0.33	-2.59 ± 0.37	n.s.	-2.65 ± 0.37	-2.84 ± 0.37	0.004
FIB-4 index	5.99 ± 3.50	5.14 ± 3.07	0.035	4.25 ± 2.47	3.19 ± 1.50	<0.001
Metabolites						
Methionine (μg/mL)	3.99 ± 1.75	4.06 ± 1.16	n.s.	3.00 ± 1.67	3.58 ± 1.38	0.012
Met-sulfoxide (x10^2ng/mL)	0.66 ± 0.30	0.57 ± 0.64	n.s.	3.30 ± 4.70	2.01 ± 3.17	0.004
Hydroxyproline (μg/mL)	1.67 ± 0.69	1.83 ± 0.67	n.s.	1.53 ± 0.64	1.71 ± 0.85	n.s.
Carnitine (μg/mL)	6.60 ± 1.41	6.83 ± 1.42	n.s.	11.90 ± 6.78	11.95 ± 6.98	n.s.
Ornithine (μg/mL)	23.26 ± 5.26	24.37 ± 5.65	n.s.	21.20 ± 7.75	18.69 ± 8.51	0.027
ADMA (x10^2ng/mL)	1.56 ± 0.43	1.64 ± 0.31	n.s.	1.61 ± 0.31	1.63 ± 0.29	n.s.
Arginine (μg/mL)	15.88 ± 4.78	16.22 ± 3.89	n.s.	12.41 ± 6.95	12.44 ± 7.20	n.s.
Acetyl-carnitine (μg/mL)	2.21 ± 2.49	2.32 ± 2.46	n.s.	2.40 ± 1.53	2.85 ± 1.60	0.007
MSR	0.19 ± 0.15	0.16 ± 0.24	n.s.	3.54 ± 13.43	0.66 ± 1.06	<0.001
OAR	1.64 ± 0.80	1.62 ± 0.72	n.s.	2.30 ± 1.63	1.94 ± 1.01	n.s.

Table S8. Characteristics of the enrolled patients with or without HCC development after achieving SVR. Data are presented as mean (±SD) unless otherwise indicated. Mann–Whitney U test; ns, not significant. Abbreviations: ASV, asunaprevir; BMI, body mass index; DCV, daclatasvir; HCV, hepatitis C virus; HCC, hepatocellular carcinoma; GLE, glecaprevir; IFN, interferon; LDV, ledipasvir; PIB, pibrentasvir; Pre-Tx, pre-treatment; SOF, sofosbuvir.

Factors	HCC	non-HCC	*p*-value
Patients, n	29	58	
Age (yr)	73 ± 6	73 ± 6	n.s.
Sex (male, n (%))	11 (37.9)	25 (43.1)	n.s.
BMI (kg/m^2)	22.4 ± 3.7	21.4 ± 3.0	n.s.
Alcohol intake ≥20g/day, n(%)	2 (6.9)	3 (5.2)	n.s.
Diabetes mellitus, n (%)	11 (37.9)	8 (13.8)	0.010
Past history of IFN treatment, n (%)	10 (34.5)	24 (41.4)	n.s.
Past history of HCC, n (%)	8 (27.6)	6 (10.3)	0.040
HCV serogroup1, n (%)	29 (100)	53 (91.4)	n.s.
Treatment, n (%)			
DCV/ASV	13 (44.8)	36 (62.1)	n.s.
SOF/LDV	12 (41.4)	13 (22.4)	n.s.
GLE/PIB	3 (10.3)	9 (15.5)	n.s.
Others	1 (3.4)	0 (0)	n.s.
Pre-Tx HCV titer (Log IU/mL)	5.7 ± 0.7	5.8 ± 0.9	n.s.

Table S9. Serum metabolites and liver function tests of patients with or without HCC development after achieving SVR. The metabolite data are the same as that in Figure 3. Data are presented as mean (±SD). Wilcoxon signed-rank test; ns, not significant. Abbreviations: AFP, alpha-fetoprotein; ALBI, albumin–bilirubin; ALT, alanineaminotransferase; AST, aspartateaminotransferase; FIB-4, fibrosis-4; HCC, hepatocellular carcinoma; MSR, methionine–methionine sulfoxide ratio; Pre-Tx, pretreatment; Post-Tx, post-treatment; SVR, sustained virological response.

**Factors**	**HCC**			**non-HCC**		
	**Pre-Tx**	**Post-Tx**	***p*-value**	**Pre-Tx**	**Post-Tx**	***p*-value**
Liver function tests						
AST (U/L)	54 ± 25	25 ± 5	<0.001	50 ± 31	25 ± 6	<0.001
ALT (U/L)	46 ± 27	18 ± 6	<0.001	42 ± 27	16 ± 10	<0.001
Platelet counts (x10^9/L)	101 ± 41	113 ± 45	0.015	143 ± 51	153 ± 49	0.004
T. Bilirubin (mg/dL)	0.9 ± 0.3	1.0 ± 0.4	n.s.	0.8 ± 0.3	0.9 ± 0.3	n.s.
Albumin (g/dL)	3.9 ± 0.4	4.1 ± 0.4	0.008	4.0 ± 0.4	4.3 ± 0.3	<0.001
AFP (ng/mL)	17.3 ± 20.6	7.8 ± 5.1	<0.001	10.5 ± 19.9	3.9 ± 2.6	<0.001
ALBI score	-2.53 ± 0.36	-2.69 ± 0.34	0.018	-2.68 ± 0.37	-2.87 ± 0.29	<0.001
FIB-4 index	6.67 ± 3.24	4.46 ± 1.71	<0.001	4.78 ± 3.16	3.57 ± 1.66	<0.001
Metabolites						
Methionine (μg/mL)	4.23 ± 1.25	3.86 ± 1.24	n.s.	2.64 ± 1.56	3.59 ± 1.75	<0.001
Met-sulfoxide (x10^2ng/mL)	3.48 ± 3.76	3.21 ± 3.38	n.s.	3.63 ± 5.29	1.63 ± 2.71	<0.001
Ornithine (μg/mL)	22.41 ± 6.88	21.09 ± 7.15	n.s.	18.98 ± 7.50	16.50 ± 7.83	0.011
Acetyl-carnitine (μg/mL)	3.49 ± 1.76	4.33 ± 2.04	0.007	2.53 ± 1.86	3.12 ± 2.20	<0.001
MSR	0.10 ± 0.15	0.12 ± 0.21	n.s.	0.64 ± 2.74	0.06 ± 0.10	<0.001

Table S10. Apparent and optimism-corrected C-index values for clinically established and metabolite-based Cox prediction models. The apparent C-index represents the discrimination performance estimated in the original study cohort. Optimism-corrected C-index values were obtained using bootstrap resampling (B = 1000) to adjust for model optimism and provide an internally validated estimate of model discrimination. Abbreviations: AFP, alpha-fetoprotein; FIB-4, fibrosis 4; Met, methionine; MetO, methionine sulfoxide; Orn, ornithine; Post-Tx, post-treatment; Pre-Tx, pre-treatment.

	model	apparent C-index	optimism-corrected C-index
Pre-Tx	FIB-4	0.704	0.702
	AFP	0.708	0.701
	FIB-4+AFP	0.724	0.707
	FIB-4+Met	0.783	0.775
	FIB-4+Orn	0.714	0.702
	FIB-4+Met+Orn	0.773	0.758
Post-Tx	FIB-4	0.649	0.646
	AFP	0.757	0.758
	FIB-4+AFP	0.752	0.743
	FIB-4+MetO	0.689	0.678
	FIB-4+Orn	0.717	0.705
	AFP+MetO	0.764	0.76
	AFP+Orn	0.782	0.777
	MetO+Orn	0.714	0.706
	FIB-4+MetO+Orn	0.742	0.725
	AFP+MetO+Orn	0.801	0.794

Reference

36 Caviglia, G.P.; Troshina, G.; Santaniello, U.; Rosati, G.; Bombaci, F.; Birolo, G.; Nicolosi, A.; Saracco, G.M.; Ciancio, A. Long-Term Hepatocellular Carcinoma Development and Predictive Ability of Non-Invasive Scoring Systems in Patients with HCV-Related Cirrhosis Treated with Direct-Acting Antivirals. Cancers 2022, 14, 828. https://doi.org/10.3390/cancers14030828.
